# CED4 and CED4-like Peptides as Effective Plant Parasitic Nematicides

**DOI:** 10.3390/molecules30183790

**Published:** 2025-09-18

**Authors:** Alejandro Calderón-Urrea, Aksa Antony Elavinal, Venu Polineni, Glenda W. Polack, Sopanha Peo

**Affiliations:** 1 Department of Biology, California State University, Fresno, CA 93740, USA; akszshine15@gmail.com (A.A.E.); sopanha_peo@mail.fresnostate.edu (S.P.); 2 Telluris Biotech India Pvt. Ltd., Hyderabad 500 033, India; vpolineni@tellurisbiotech.com; 3 Telluris Envirotechnology, Fresno, CA 93711, USA; glendapolack@gmail.com

**Keywords:** root-knot nematodes, *Caenorhabditis elegans*, *Meloidogyne incognita*, plant parasitic nematodes, CED4, peptides

## Abstract

Plant parasitic nematodes are a significant agricultural threat, causing substantial economic losses. Methyl bromide, a commonly used nematicide, has been banned due to its harmful environmental and human health effects. As an alternative, the expression of the programmed cell death (PCD) gene CED4 from *Caenorhabditis elegans* in transgenic plants has been proposed to control nematode populations. In this study, the interaction between CED4 and other proteins was analyzed, and peptide sequences representing interaction domains were identified. Efficacy assays demonstrated that specific peptides—particularly Peptides 2 and 3 (N-terminal α/β domain) and Peptide 12 (C-terminal HD-2 domain)—induced significant mortality in *C. elegans*, while other peptides were ineffective. The study further investigated whether these peptides, along with modified CED4-like peptides (2a, 3a, and 12a), induce PCD in *C. elegans* via the activation of the nematode’s endogenous PCD pathway. Testing was conducted on wild-type and mutant strains of *C. elegans* (*ced-4* and *ced-3* mutants). Nematode survival was monitored over 34 days, revealing that *c3* mutants survived exposure to CED4-like peptides, suggesting that the peptides trigger PCD through the activation of the endogenous cell death pathway. These findings support the potential use of CED4-based peptides as a novel strategy for nematode control.

## 1. Introduction

Plant-parasitic nematodes (PPNs) represent a formidable threat to global agricultural productivity, causing estimated annual losses of approximately USD 157 billion dollars [1]. Among these, *Meloidogyne incognita* (*M. incognita*), a root-knot nematode capable of infecting over 2000 plant species, stands out as a particularly destructive pathogen. Traditional control measures, including the application of chemical nematicides such as methyl bromide, have provided effective solutions in the past. However, their continued use has raised serious environmental and health concerns. Methyl bromide, for instance, has been banned in many countries due to its ozone-depleting properties and toxicological effects on humans and ecosystems [2]. Consequently, the search for sustainable, ecologically sound, and economically viable alternatives for PPN control has become a pressing research priority.

Recent advances in molecular biology and biotechnology offer promising avenues for nematode control. In particular, the model organism *Caenorhabditis elegans* (*C. elegans*), due to its simple anatomy, well-characterized genome, and conserved programmed cell death (PCD) pathway, has served as a valuable system for understanding the molecular mechanisms underlying nematode biology. Programmed cell death in *C. elegans* is orchestrated by a genetic pathway involving core proteins such as CED3, CED4, and CED9, which coordinate apoptotic responses that are essential for development and homeostasis [3,4]. CED4, a 549-residue protein encoded by the CED4 gene, plays a pivotal role in the activation of CED3, the caspase enzyme responsible for executing cell death. Structural studies have shown that CED4 forms an apoptosome complex that is crucial for the activation of downstream apoptotic cascades.

In this context, we pioneered the exploration of using PCD-related proteins and their derivatives as novel nematicidal agents. Preliminary investigations demonstrated that recombinant CED4 protein and synthetic chalcone derivatives exhibit potent nematocidal effects against both *C. elegans* and *M. incognita* under laboratory conditions [5,6]. Moreover, specific peptide segments derived from the CED4 protein were found to induce nematode mortality at low concentrations (identified here as Peptides 2, 3, and 12), suggesting their potential as effective and environmentally safe biocontrol agents [7].

This study builds on those foundational findings with the aim of elucidating the mechanistic basis of nematode death induced by CED4-derived peptides. Specifically, we hypothesize that certain minimal peptide domains from the CED4 protein, including both native and CED4-like variants (containing neutral amino acid substitutions), can trigger the endogenous PCD pathway in *C. elegans*. To test this, we assessed nematode mortality in wild-type as well as *ced-3* and *ced-4* mutant strains of *C. elegans* following peptide exposure. Demonstrating that these peptides can initiate apoptosis via the nematode’s intrinsic cell death machinery would not only validate their functional specificity but also support their potential utility as biopesticides in sustainable agriculture.

## 2. Results

### 2.1. CED4 Peptide Design and Testing

**Figure 1:**
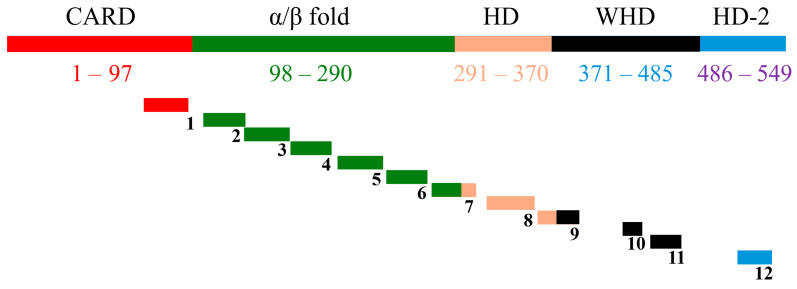
Diagrammatic view of full CED4 along with the 12 designed peptides. Shows the full CED4 along with the domains (color coded) and the corresponding position of the designed peptides on CED4 domains (not to scale). Figure 7 from [7].

Previous studies employing transgenic plants expressing the *C. elegans* programmed cell death gene *ced-4* demonstrated that programmed cell death (PCD) regulators, particularly CED4, could be harnessed as an effective strategy to control plant-parasitic nematodes [6]. Building on this concept, a panel of twelve synthetic peptides was rationally designed from the full-length CED4 protein sequence (Figure 1, Table 1) [7]. The design process incorporated four critical bioinformatic and structural criteria: (1) residue conservation within the CED4 protein, assessed using HotSpot Wizard to predict functionally tolerant mutation sites [8]; (2) inter-residue interactions with other apoptosis-related proteins such as CED3, CED9, and ATP-binding residues, identified through PDBsum interaction maps [9]; (3) preservation of secondary structural motifs, determined using consensus predictions from the 2Struc server [10]; and (4) the surface accessibility of peptide regions, evaluated using Superficial software to ensure presentation on the protein surface [11].

Each synthesized peptide was tested for nematocidal activity against wild-type *C. elegans* and the second-stage juveniles (J2) of *Meloidogyne incognita*. Peptide exposure was conducted in 96-well microtiter plates, where each well represented an independent replicate and contained nematodes suspended in peptide solution at a final concentration of 0.8 mg/mL. For each peptide, nine biological replicates were performed. A parallel series of assays was also conducted at 0.4 mg/mL (see Figures S1 and S2 for details).

**Figure 2:**
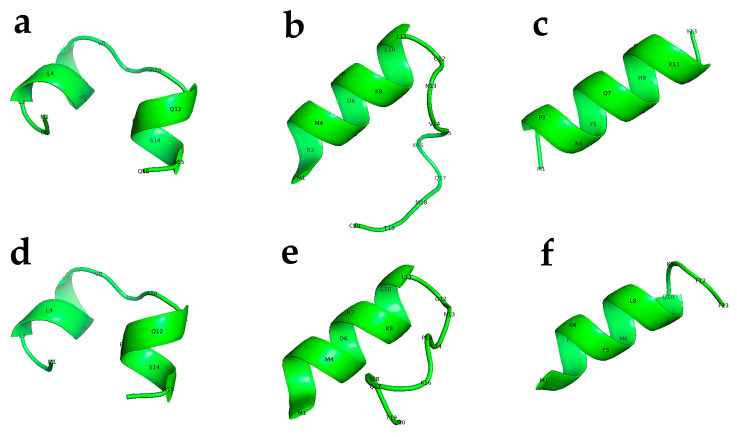
Predicted 3D structure for the active peptides used in the current study. CED4 Peptides 2 (**a**), Peptide 3 (**b**), Peptide 12 (**c**), and CED4-like Peptide 2a (**d**), Peptide 3a (**e**), and Peptide 12a (**f**). These 3D structure predictions were generated with PEP-FOLD3.5 and rendered into TIFF files using PyMOL (The PyMOL Molecular Graphics System, Version 1.2r3pre, Schrödinger, LLC. New York, NY, USA).

Among the twelve peptides tested, three peptides—Peptide 2, Peptide 3, and Peptide 12—elicited complete lethality (100%) in both *C. elegans* and *M. incognita* J2s (see Figure 2 for a 3D model structure of these peptides). In contrast, the remaining peptides exhibited minimal or no lethality under identical assay conditions. To further assess the functional robustness of the active peptides, analogs of Peptides 2, 3, and 12 were synthesized with two amino acid substitutions each, yielding CED4-like variants designated as Peptide 2a, Peptide 3a, and Peptide 12a (Table 1). These modified peptides retained high nematocidal efficacy, inducing mortality, although at lower levels comparable to their unmodified counterparts. The results suggest that minor sequence alterations do not abolish the biological activity of these peptides, highlighting their potential utility as broad-spectrum nematicides [7].

### 2.2. Confirmation of the Effects of CED4 and CED4-like Peptides on Wild-Type C. elegans Using Lifespan Assays

We first sought to confirm that the CED4 and CED4-like peptides indeed induce mortality in *Caenorhabditis elegans* by performing a lifespan assay, a well-established method for assessing compound effects on nematode longevity. This approach has been extensively validated in prior studies utilizing *C. elegans* as a model for toxicological screening and survival analysis [12,13]. To this end, we tested the effects of CED4-derived Peptides 3 and 12, as well as their analogs—CED4-like Peptides 3a and 12a—at a concentration of 0.8 mg/mL. In addition, we examined the potential for synergistic activity by testing combinations of Peptides 3 + 12 and 3a + 12a.

**Figure 3:**
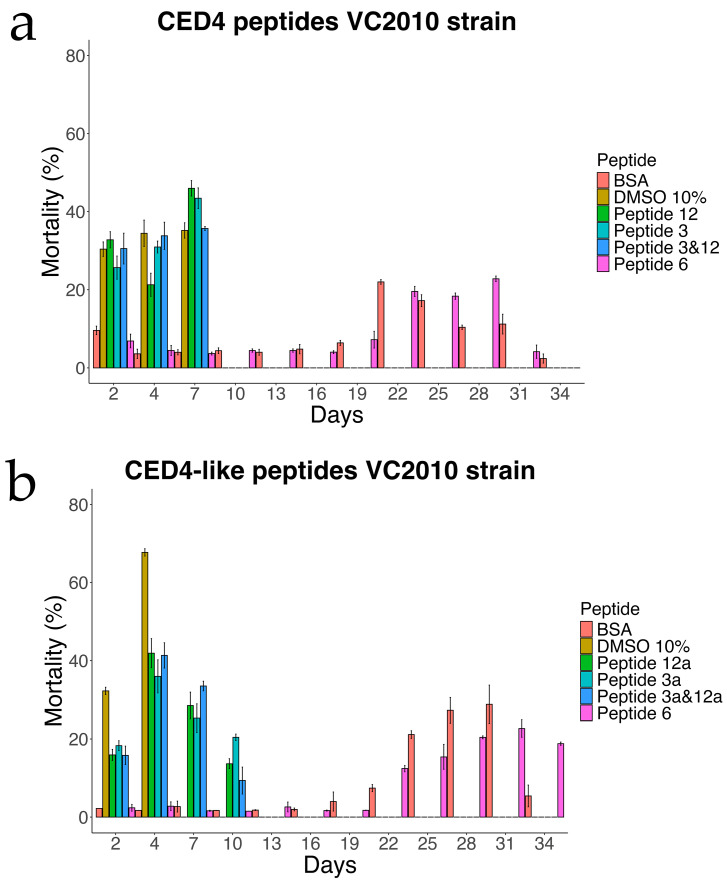
Experiment of Individual CED4 peptides 3, 12 and combination of 3 and 12 (**a**) and CED4-like peptides 3a, 12a and combination of 3a and 12a (**b**), at a concentration of 0.8 mg/mL for VC2010 strain of *C. elegans*.

Each experimental condition included appropriate controls: bovine serum albumin (BSA) and Peptide 6 served as negative controls, while 10% dimethyl sulfoxide (DMSO) was employed as a positive control. DMSO is known to reduce the lifespan of *C. elegans* at concentrations exceeding 5%, thereby validating its use as a cytotoxic reference compound [14]. As shown in Figure 3, the results corroborated our prior findings [7] and confirmed that Peptides 3 and 12 independently induce mortality in *C. elegans*, whereas Peptide 6 exhibited no lethality and thus was reaffirmed as a reliable negative control.

Importantly, while previous evaluations were based on a binary dead-versus-alive scoring metric, the current study employs quantitative lifespan assays, providing a more nuanced assessment of peptide-induced effects. This methodology also enabled an exploration of potential combinatorial synergy among CED4 peptides. Accordingly, we designed an assay to test all 66 possible pairwise combinations of the twelve CED4 peptides at a total peptide concentration of 0.4 mg/mL (see Figure S1 for details). Peptide 6 was used as the negative control, and Peptide 12 served as a positive control. A follow-up experiment was conducted at a higher concentration of 0.8 mg/mL to validate and extend the findings from the initial screen (see Figure S2 for details).

### 2.3. Synergistic Effects of CED4 Peptide Combinations on C. elegans Mortality

To investigate the potential synergistic effects among the twelve designed CED4 peptides, a comprehensive series of combinatorial assays was conducted at two total concentrations: 0.4 mg/mL and 0.8 mg/mL. Each peptide was systematically paired with the remaining eleven to yield 66 unique combinations per concentration, and their impacts on nematode mortality and apoptosis induction were assessed over a 96 h period.

At 0.4 mg/mL, notable synergy was observed in several pairings (Figure S1). Peptide 1 demonstrated heightened nematocidal efficacy when combined with Peptides 2, 3, and 12, with the Peptide 1 + 2 combination achieving nearly 60% mortality. Peptide 2 exhibited induced apoptosis nearing 20% at 96 h when paired with other peptides, particularly with Peptide 3, which yielded the highest efficacy among combinations. Peptide 4, although generally less effective, surpassed 30% efficacy when combined with Peptides 2, 3, or 12. In contrast, Peptides 5 and 6 displayed minimal apoptotic or lethal effects, with Peptide 6 serving reliably as a negative control due to its consistently low activity.

Peptide 7 showed modest nematode mortality (~10%) in combinations with several peptides, while Peptide 8 demonstrated apoptosis rates of ≥30% with Peptides 2, 3, and 12. Peptide 9, when combined with Peptide 12, showed elevated mortality rates, and combinations involving Peptides 10 and 11 exhibited mild synergistic effects, particularly with Peptide 12. Notably, Peptide 12 displayed robust performance, with combinations across most peptides producing mortality rates >40% by Day 4, indicating a general synergistic enhancement in apoptosis induction.

At the higher concentration of 0.8 mg/mL, the synergistic effects were amplified (Table 2 and Figure S2). The combination of Peptide 1 with Peptides 2, 3, and 12 achieved complete (100%) nematode mortality by Day 4. Peptide 2, when paired with all other peptides, consistently exceeded 70% mortality, confirming its broad synergistic potential. Combinations involving Peptide 3 also showed significant efficacy, with some pairings (e.g., with Peptides 1, 2, 4, 5, 7, 9, and 12) reaching 100% mortality. Even peptides previously classified as negative (e.g., 6 and 10) demonstrated mortality at >60% when paired with active peptides.

Further, Peptide 4 exhibited rapid efficacy, with complete nematode death achieved within 72 h when paired with Peptide 12. Peptides 5 and 12 were particularly potent in combination with Peptide 3, while Peptide 6 showed selective activity, particularly with Peptides 2 and 3. Peptide 7 combinations induced apoptosis levels above 25% in several pairings, including with Peptides 4, 6, 8, 9, and 11. Similarly, Peptide 8 showed enhanced apoptosis in combinations with Peptides 2, 3, and 12.

Peptides 10 and 11, although less consistently effective, demonstrated increased mortality in select pairings—especially Peptide 11 with Peptides 2, 3, and 12, reaching up to 70%. Peptide 12, the most potent among all, induced nearly 100% mortality within 72 h when combined with Peptide 3, and retained >80% efficacy in multiple other pairings, including those with peptides previously deemed less active.

These findings underscore the concentration-dependent synergistic interactions among specific CED4 peptide combinations, with several pairs—most notably those involving Peptides 1, 2, 3, and 12—exhibiting pronounced nematocidal and apoptotic effects. The results validate the strategy of combinatorial peptide application for enhancing programmed cell death in nematodes.

### 2.4. CED4 Peptides Induce Differential Responses in Mutant ced-3 and ced-4 Nematodes

**Figure 4:**
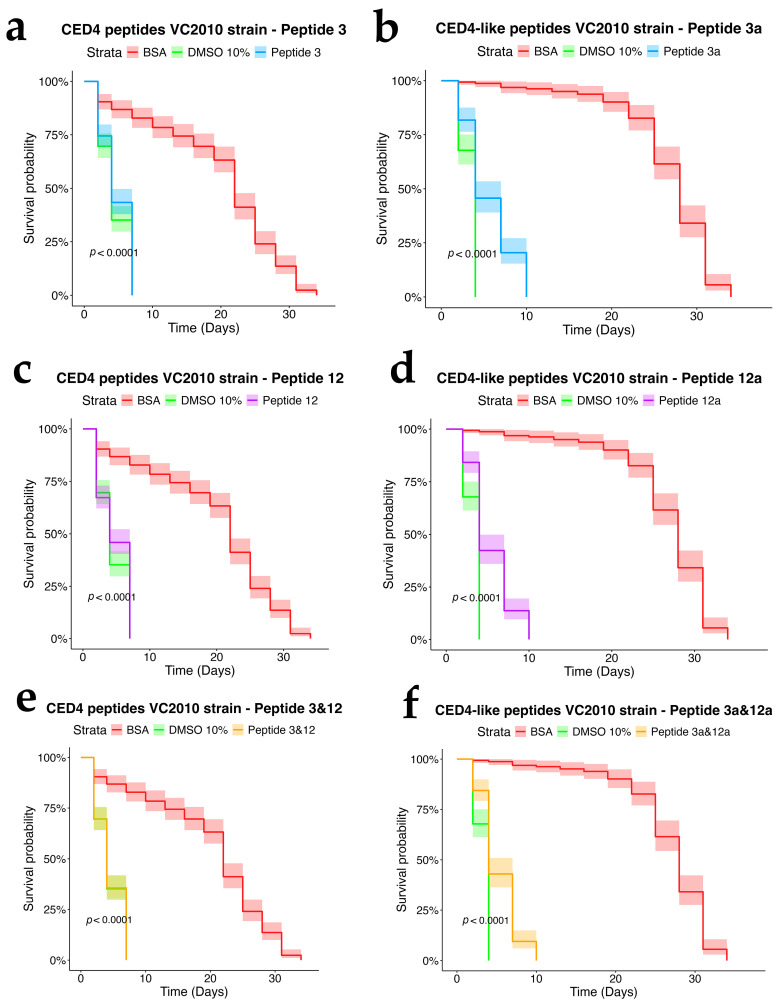
Survival chart of individual CED4 peptides 3 (**a**), 12 (**c**), and combination of 3 and 12 (**e**), and CED4-like peptides 3a (**b**) and 12a (**d**) and combination of 3a and 12a (**f**), at a concentration of 0.8 mg/mL for VC2010 strain of *C. elegans*.

**Figure 5:**
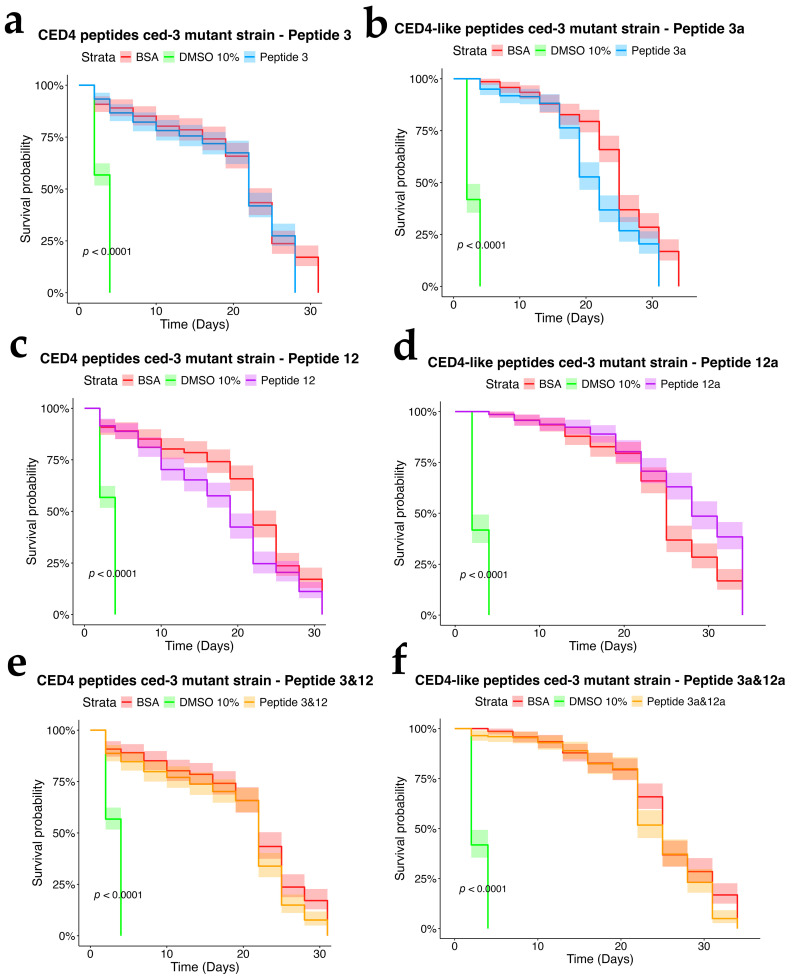
Survival charts of *C. elegans ced-3* mutant exposed to individual CED4 peptides 3 (**a**), 12 (**c**), combination of 3 and 12 (**e**), and CED4-like peptides 3a (**b**) and 12a (**d**) and combination of 3a and 12a (**f**), at a concentration of 0.8 mg/mL.

**Figure 6:**
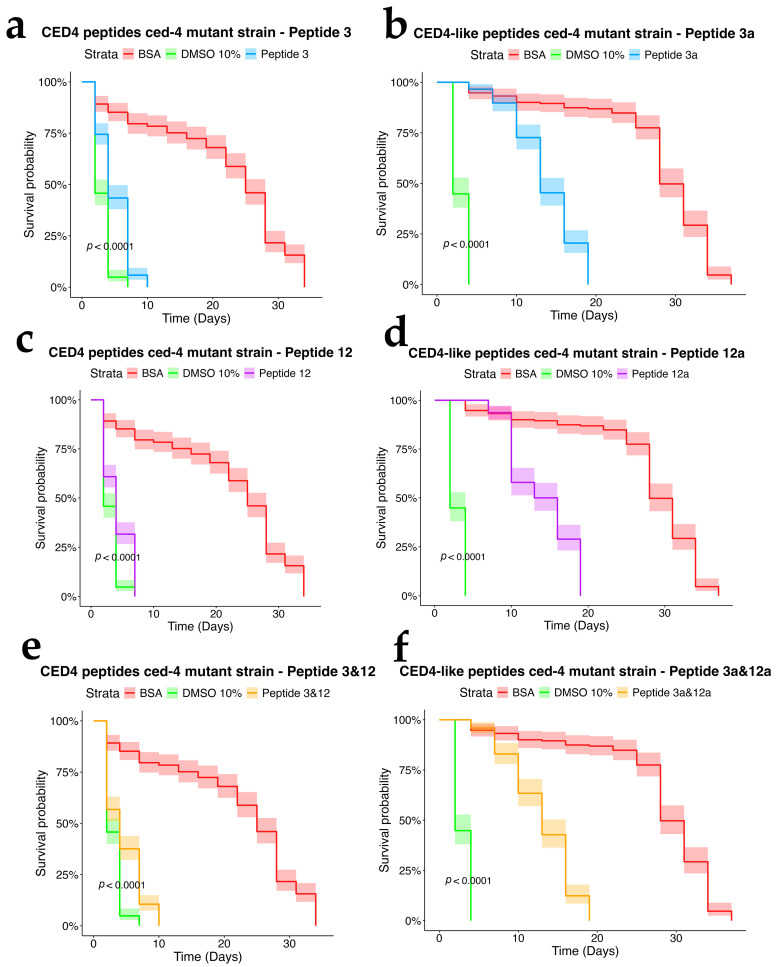
Survival charts of *C. elegans ced-4* mutant exposed to individual CED4 peptides 3 (**a**), 12 (**c**), combination of 3 and 12 (**e**), and CED4-like peptides 3a (**b**) and 12a (**d**) and combination of 3a and 12a (**f**), at a concentration of 0.8 mg/mL.

To assess the dependency of CED4 peptide-induced mortality on apoptotic signaling, we tested Peptides 3 and 12, as well as their combination, on *ced-3* (*n717*) and *ced-4* (*n1162*) mutant strains of *C. elegans*, alongside wild-type controls. Each treatment was conducted at a concentration of 0.8 mg/mL, and the results were evaluated using a survival analysis over a 34-day period (Figure 4, Figure 5 and Figure 6). In both mutant strains (Figure 5 and Figure 6), BSA-treated controls exhibited high survival rates, with over 80% of individuals alive at day 34, confirming the lack of cytotoxicity from the vehicle. Conversely, worms exposed to 10% DMSO began dying by Day 2, with full mortality observed by Day 5. This pattern was consistent across all three biological replicates.

Peptide 12 elicited strong lethality in *ced-4* mutants, and the combination of Peptides 3 and 12 further accelerated death, with complete mortality occurring by Day 9. Notably, no significant mortality was observed in *ced-3* mutants until after Day 24, and survival in these groups was comparable to the BSA control through most of the lifespan assay. These data suggest that CED4 peptides mediate programmed cell death primarily through a *ced-3*-dependent pathway. The lack of response in *ced-3* mutants and pronounced mortality in *ced-4* mutants support the hypothesis that exogenous CED4 peptides induce the deaths of nematodes by acting on the endogenous PCD of the nematode.

### 2.5. Assessment of CED4-like Peptides on Wild-Type and Mutant Strains

We further evaluated the biological effects of synthetic CED4-like peptides (3a and 12a) on wild-type VC2010, *ced-3*(*n717*), and *ced-4*(*n1162*) mutant *C. elegans* strains using lifespan assays at a peptide concentration of 0.8 mg/mL (Figure 4, Figure 5 and Figure 6). As with previous experiments, BSA and DMSO served as negative and positive controls, respectively.

In the wild-type strain (Figure 4), both Peptides 3a and 12a exhibited time-dependent nematocidal activity. Peptide 12a caused complete mortality by Day 18, while the combination of Peptides 3a and 12a accelerated lethality, achieving total death by Day 16. This outcome supports a synergistic effect of the two peptides. In *ced-3* mutants (Figure 5), no substantial mortality was observed in response to CED4-like peptides before Day 24, with survival curves resembling those of the BSA control. In contrast, *ced-4* mutants (Figure 6) displayed significant sensitivity to both individual and combined CED4-like peptides, with most individuals dying between Days 18 and 20, suggesting partial activation of the programmed cell death pathway.

Although the CED4-like peptides triggered cell death in *C. elegans*, the onset of lethality was notably delayed compared to CED4 peptides. Furthermore, Peptides 3a and 12a exhibited lower solubility and increased viscosity in M9 buffer, complicating microscopic observation and accurate survival scoring in 96-well assays. 

### 2.6. Comparative Analysis of CED4 and CED4-like Peptide Activity

Collectively, these results indicate that while both CED4 and CED4-like peptides induce nematode death through the endogenous apoptotic machinery, the canonical CED4 peptides (Peptides 3 and 12) demonstrate more rapid and potent effects across wild-type and mutant strains. The CED4-like peptides (3a and 12a) exhibited delayed activity and reduced efficacy, likely due in part to their physicochemical properties, including reduced solubility. These findings reinforce the potential of CED4 peptides as efficient, targeted nematicidal agents with a well-defined mode of action based on apoptotic pathway activation.

## 3. Discussion

The urgent need for environmentally sustainable alternatives to conventional chemical nematicides has driven the exploration of peptide-based biopesticides, which are generally recognized by regulatory bodies such as the EPA as eco-friendly solutions. Several recent studies have demonstrated the potent nematocidal properties of naturally derived peptides. For instance, rhabdopeptides from *Xenorhabdus budapestensis* SN84 exhibit high toxicity against second-stage juveniles (J2) of *Meloidogyne incognita* [15]. Similarly, lipopeptides such as thumolycin from *Bacillus thuringiensis* (Bt) and Bt-derived crystal proteins (Cry5B, Cry6A, Cry14A, Cry21A) have shown efficacy in killing *Caenorhabditis elegans* and other nematode species through mechanisms involving neurotoxicity [16,17]. Additional peptides, including neurotoxins from *Androctonus australis* and peptaibols from *Ijuhya vitellina*, have demonstrated variable efficacy across nematode and insect targets, with some compounds showing enhanced activity when combined with entomopathogenic fungi [18,19].

In addition to the Bt-derived crystal proteins and the vegetative insecticidal proteins (Vip), which are produced during the vegetative growth phase and are considered an excellent toxic candidate because of the difference in sequence homology and receptor sites from Cry proteins [20], Gram-negative bacteria and entomopathogenic fungi have also been the subject of in-depth research on insectotoxic proteins. These examples offer crucial background information for our findings. For example, Beauvericin, a cyclodepsipeptide with potent insecticidal activity against lepidopteran larvae, is produced by the entomopathogenic fungus *Beauveria bassiana* [21]. Similarly, destruxins, a class of cyclic hexadepsipeptides that upset ion balance and paralyze insects, are produced by *Metarhizium anisopliae* [22]. Tc (toxin complex) proteins, which are strong oral insecticides that disrupt the cytoskeleton and kill midgut cells, are secreted by *Photorhabdus luminescens*, a Gram-negative bacterium [23]. Additionally, xenocin and related bacteriocin-like proteins with insecticidal qualities are produced by *Xenorhabdus nematophila* [24]. Furthermore, it has been demonstrated that *Serratia entomophila* produces the Sep toxin complex, which is exclusively directed against grass grub larvae [25]. More recently, new insecticides have been successfully developed from spider-venom peptides (see, for example [26,27]) providing new alternatives to the pesticide control of insects. Gram-negative bacteria, fungi and spiders can produce insectotoxic proteins in a variety of ways, as demonstrated by these examples, underscoring their ecological significance and potential as biocontrol agents. However, and to our knowledge, no other peptide, beyond the ones described here, has been reported with nematicidal activity targeting the PCD endogenous pathway.

In this context, the present study expands the application of peptide biopesticides by examining CED4-derived and CED4-like peptides, which were rationally designed based on the apoptotic regulator CED4 of *C. elegans*. The results reveal that specific peptides—particularly Peptides 2, 3, and 12—induce complete lethality in both *C. elegans* and *M. incognita*, indicating strong nematocidal activity. These findings support the hypothesis that the mode of action of these peptides involves the activation of the nematodes’ endogenous programmed cell death (PCD) pathways.

Importantly, combinations of these peptides revealed a synergistic enhancement in nematocidal efficacy, particularly for Peptides 3 and 12, as well as their CED4-like analogs 3a and 12a. These analogs, containing only minor amino acid substitutions, retained full activity, suggesting that the apoptotic mechanism is robust to minor sequence variation. This is consistent with the structural role of CED4 in apoptosis, which appears to be conserved across nematode taxa.

**Figure 7:**
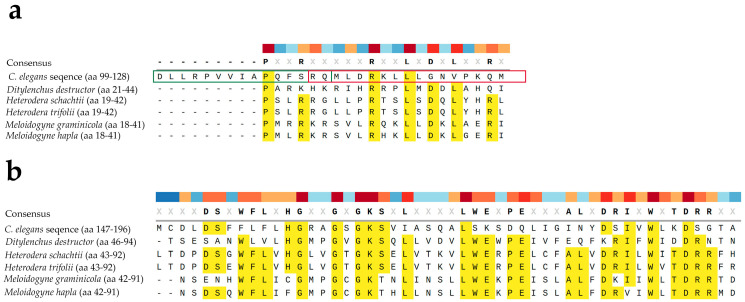
Multiple alignment between two sections (**a**,**b**) of CED4 from *C. elegans* (GenBank accession number C35D10.9a.1) and putative homologs from *Ditylenchus destructor* (GenBank accession number KAI1726433.1), *Heterodera schachtii* (GenBank accession number KAL3102088.1), *Heterodera trifolii* (GenBank accession number KAL3110170.1), *M. graminicola* (GenBank accession number KAF7640432.1), and *M. hapla* (GenBank accession number KAL7072841.1). Conserved amino acids (aa) are highlighted in yellow; other aa seem to be variable. In (**a**) the alignment is around the Peptide 2 (green box) and Peptide 3 (red box). The numbers in parenthesis next to the scientific names indicate the corresponding amino acids (aa) in the protein. Multiple alignment and visualization were conducted using SnapGene software (www.snapgene.com).

The conservation of apoptotic pathways among nematodes was further explored through bioinformatic comparison of the CED4 protein sequence with entries in NCBI GenBank. Homologous sequences were found not only in several *Caenorhabditis* species (e.g., *C. briggsae*, *C. remanei*, *C. nigoni*, *C. bovis*) but also in plant-parasitic and animal-parasitic nematodes. Figure 7 shows alignment between CED4 from *C. elegans* and putative homologs from *Ditylenchus destructor*, *Heterodera schachtii*, *Heterodera trifolii*, *M. graminicola*, and *M. hapla*. A one-on-one comparison between *C. elegans* and *M. hapla* shows 18% identity and 32% positive substitutions. Similarly, a one-on-one comparison between *C. elegans* and *D. destructor* shows 17% identity and 32% positive substitutions. Despite their taxonomic differences, many of these nematodes belong to the superfamily Tylenchoidea, suggesting that a conserved apoptotic machinery may be a shared vulnerability across species. Our focus was on sedentary phytonematodes as they are the most economically significant group of plant parasitic nematodes and their genomes are better characterized.

These findings parallel reports in bacterial systems, where conserved genes across diverse species of *Nocardiopsis* mediate essential survival functions despite genetic divergence [28].

Interestingly, weak homologies to the CED4 sequence were observed in some soil microorganisms, such as *Choanephora cucurbitarum*, *Blakeslea trispora*, and *Rhizophagus irregularis*. Although no definitive conclusions can yet be drawn regarding functional similarities, these observations warrant further investigation into potential off-target effects and the evolutionary context of apoptotic signaling in non-metazoan species.

Taken together, the data suggest that CED4 peptides, particularly Peptides 3 and 12 and their analogs, are promising candidates for the development of novel peptide-based nematicides. Their mechanism—targeting an essential and highly conserved endogenous cell death pathway—confers specificity and reduces the likelihood of resistance development. This is particularly important given the increasing reports of resistance to conventional nematicides in agricultural systems. Furthermore, the evidence of structural and functional conservation across plant- and animal-parasitic nematodes highlights the broad applicability of these peptides.

Preliminary works [29] as well as future works should focus on the field-based validation of peptide efficacy in diverse agricultural contexts and expanded sequence homology studies across additional nematode genera. In parallel, the structural studies of peptide–nematode interactions and apoptotic cascade activation will further elucidate the precise molecular mechanisms underlying nematode death. These efforts will ultimately inform the rational design of second-generation peptide-based nematicides with optimized stability, delivery, and specificity.

## 4. Materials and Methods

### 4.1. Nematode Strains and Maintenance

The *Caenorhabditis elegans* strains used in this study included the wild-type VC2010, the *ced-3 (n717)* mutant, and the *ced-4 (n1162)* mutant. All strains were obtained from the Caenorhabditis Genetics Center (CGC https://cgc.umn.edu/ accessed on 16 July 2025), which is funded by the NIH Office of Research Infrastructure Programs (P40 OD010440). Nematodes were maintained on nematode growth medium (NGM) agar plates seeded with *Escherichia coli* OP50, as described previously [30]. Plates were incubated at 20 °C and transferred weekly to fresh OP50-seeded NGM plates to maintain healthy populations.

### 4.2. Peptide Design and Preparation

Twelve peptide segments derived from the CED4 protein were designed using in silico techniques, with each peptide ranging from 12 to 25 amino acids in length. Peptide design was guided by four parameters: (1) the conservation of active-site residues using HotSpot Wizard [8], (2) the structural and binding interaction data from PDBsum with ATP, CED3, CED4, and CED9 [9], (3) the inclusion of complete secondary structure elements obtained from the 2Struc secondary structure consensus server [10], and (4) the surface accessibility analysis using the Superficial software tool [5]. Final peptide selections were based on a comparison of designed peptides with surface-accessible consensus segments. Peptides were synthesized by Genscript (Piscataway, NJ, USA); see Supplemental Materials for relevant synthesis data Peptides 2, 2a, 3, 3a, 12, 12a, and 6.

### 4.3. Synchronization of Worm Populations

Adult nematodes were synchronized using an established bleaching protocol [31]. Worms were harvested from NGM plates with 2 mL of distilled water, centrifuged at 1200 rpm for 2 min, and washed with M9 buffer. Bleaching was performed using a freshly prepared solution containing 2.5 mL bleach, 0.5 mL 10 M NaOH, and 7 mL distilled water, followed by vortexing and a 4 min incubation at room temperature. Embryos were washed three times with M9 buffer and resuspended in S-complete medium, then incubated on a rotary mixer for 12–15 h to allow hatching.

### 4.4. Peptide Treatment in 96-Well Plates

Synchronized L4-stage nematodes were transferred to 96-well plates for peptide exposure. Each well contained three worms in 100 μL of peptide solution at a final concentration of 0.8 mg/mL. Peptides tested included CED4-derived peptides (3, 12) and their analogs (3a, 12a). To inhibit reproduction, 0.6 mM fluorodeoxyuridine (FUDR) was added to all wells. Worms were fed with 60 μL of OP50 suspension. Plates were sealed with parafilm and incubated at 20 °C.

### 4.5. Controls

Negative controls included Peptide 6 (a known non-toxic CED4-derived peptide) and bovine serum albumin (BSA), each prepared at 0.8 mg/mL in M9 buffer. A 10% DMSO solution (diluted in M9 buffer) served as a positive control for inducing apoptosis.

### 4.6. Viability and Survival Assessment

Worm viability was assessed every 24 h for up to 34 days. Nematodes were classified as “alive” if they responded to gentle prodding under a dissecting microscope, “dead” if unresponsive, and “censored” if survival data was ambiguous. Survival was recorded manually.

### 4.7. Experimental Replicates and Statistical Analysis

Each experimental condition was performed in triplicate, with each replicate including a minimum of 350–500 worms per 96-well plate. Across all conditions, 1000–1300 worms were tested per experiment. Survival data were analyzed using OASIS 2.0, an online platform for nematode survival analysis [12]. Data input included live, dead, and censored worm counts, and results were visualized as Kaplan–Meier graphs using R-studio [survival curves in JPEG format].

## 5. Conclusions

The present study identifies CED4-derived peptides as promising candidates for the development of environmentally safe nematicides. The CED4-derived peptides—particularly Peptides 2, 3, and 12—demonstrated high efficacy against *C. elegans* and the plant-parasitic nematode *Meloidogyne incognita* at a concentration of 0.8 mg/mL. These peptides, designed based on the conserved regions of the CED4 protein from *C. elegans*, were shown to induce mortality likely through the activation of the nematodes’ endogenous apoptotic pathways. The difference in action between Peptides 2, 3, and 12 (along with their analogs 2a, 3a, and 12a) might be due to the differences in their ability to promote the formation of functional apoptosomes in the cells of the nematodes, although this will have to be tested experimentally. Peptides 3 and 12, as well as their analogs 3a and 12a, maintained their activity despite minor amino acid substitutions, further supporting the robustness of their nematocidal mechanism.

Comparative sequence analysis revealed conserved homologs of the CED4 protein across several Caenorhabditis species and a broad spectrum of agriculturally relevant parasitic nematodes, including species of *Meloidogyne*, *Heterodera*, *Globodera*, and others. The conservation of apoptotic regulatory elements across these taxa underscores the potential for the broad-spectrum application of CED4-based peptides. Although weak sequence similarities were observed in certain soil-dwelling microorganisms such as *Choanephora cucurbitarum*, *Blakeslea trispora*, and *Rhizophagus irregularis*, further investigation is needed to assess potential non-target effects and ecological safety.

The findings presented here support the hypothesis that minimal binding domains derived from the CED4 protein are sufficient to activate lethal apoptotic pathways in nematodes, perhaps on the organs that are first exposed to the peptides such as the digestive system; in this scenario, we can envision the nematodes dying from starvation after the collapse of their digestive system. These peptides offer a promising path forward for the development of novel, target-specific nematicides. Moreover, due to their reliance on conserved, essential pathways in nematodes, the likelihood of resistance development is presumed to be low. Future studies should focus on (i) Peptides 3 and 12 (as well as Peptides 3a and 12a) on additional nematode species, (ii) testing their safety on non-target soil microbiota and beneficial organisms, and (iii) conduct additional studies validating their efficacy *in planta* under greenhouse and field conditions.

## 6. Patents

US Pat No. 9125413 B1. Nematicide composition comprising CED4 peptide. 8 September 2015. (https://patents.google.com/patent/US9125413B1/en accessed on 16 July 2025).

US Pat No. 11484035 B2 Synergistic composition of a nematicide. (1 November 2022. https://patents.google.com/patent/US11484035B2/en?oq=US+Pat+No+11%2c484%2c035+B2+ accessed on 16 July 2025).

US Pat No. 10925286 B2. Synergistic chalcone containing composition of a nematicide. 23 February 2021. (https://patents.google.com/patent/US10925286B2/en?oq=10925286 accessed on 16 July 2025).

## Figures and Tables

**Table 1:** Sequence of CED4 peptides (Peptides 1–12) and CED4-like peptides (Peptides 2a, 3a, and 12a) used in this research, along with their calculated pI and net charge at pH of 7.0 calculated using the BACHEM calculator (https://www.bachem.com/ accessed on 16 July 2025) and PepDraw (https://www2.tulane.edu/~biochem/WW/PepDraw/ accessed on 16 July 2025) from Tulane University; both sources gave the same or very similar numbers. Some of these peptides are capable of inducing death in *C. elegans* (Peptides 2, 3, 12, 2a, 3a, and 12a) and one is used as a negative control (Peptide 6) because of its inability to induce death in nematodes. The first aa of Peptides 2, 3, 12, 2a, 3a, and 12a was an added methionine. The designed peptides show a preponderance of hydrophobic residues alongside basic residues, suggesting the potential formation of amphipathic α-helices. The helical wheel projection of CED4 peptides demonstrates clear hydrophobic and hydrophilic faces, supported by a hydrophobic moment of 0.52, indicating likely membrane-interactive behavior. Such amphipathicity may facilitate insertion into nematode cuticle or root lipid interfaces, contributing to observed nematicidal activity—which is consistent with the models of membrane disruption by helical antimicrobial peptides (e.g., toroidal pore or carpet mechanisms) [12]. See Figure S3 for a predicted 3D structure for all peptides used in the current study. The asterisk represent the stop codon added to the corresponding DNA sequence of the peptide.

Item	Peptide Name and AA Sequence	pI	Net Charge (pH 7.0)
1	Peptide 1 (aa residues 80–97 from CED4): Q S H L A D F L E D Y I D F A I N E * (18)	3.77	−3.91
2	Peptide 2 (aa residues 99–113 from CED4): M D L L R P V V I A P Q F S R Q * (16)	12.49	2.00
3	Peptide 2a (aa residues 99–113 from CED4 with two changes in red): M E L L R P V V I A P Q F S R E * (16)	10.95	1.00
4	Peptide 3 (aa residues 112–130 from CED4): M R Q M L D R K L L L G N V P K Q M T C * (20)	11.53	3.95
5	Peptide 3a (aa residues 112–130 from CED4 with two changes in red): M K E M L D R K L L L G N V P K Q M T C * (20)	10.35	2.95
6	Peptide 4 (aa residues 129–141 from CED4): T C Y I R E Y H V D R V I * (13)	8.81	1.04
7	Peptide 5 (aa residues 207–220 from CED4): I L L M L K S E D D L L N F * (14)	4.40	−1.00
8	Peptide 6 (aa residues 227–240 from CED4): M T S V V L K R M I C N A L I * (15)	11.56	2.95
9	Peptide 7 (aa residues 274–294 from CED4): D V E I S N A A S Q T C E F I E V T S L E * (21)	3.39	−4.04
10	Peptide 8 (aa residues 334–358 from CED4): M M F F K S C E P K T F E K M A Q L N N K L E S R * (25)	10.18	2.96
11	Peptide 9 (aa residues 360–380 from CED4): L V G V E C I T P Y S Y K S L A M A L Q R * (21)	9.25	1.95
12	Peptide 10 (aa residues 442–456 from CED4): A L L S G K R M P V L T F K I * (15)	11.69	4.00
13	Peptide 11 (aa residues 468–484 from CED4): V D A Q T I A N G I S I L E Q R L * (17)	7.02	0.00
14	Peptide 12 (aa residues 529–540 from CED4): M F P K F M Q L H Q K F Y * (13)	10.81	3.09
15	Peptide 12a (aa residues 529–540 from CED4 with two changes in red): M Y P K F M Q L H Q K F F * (13)	10.81	3.09

**Table 2:** Qualitative description of the biological effect of the binary peptide combinations at 0.8 mg/mL. The scoring system is as follows: (−) = no effect; (+) = weak; (++) = moderate; (+++) = strong; (++++) = very strong effect. Whenever peptides 2, 3, or 12 are used in combination, there is a strong effect (red filled boxes). The only other peptide combination that showed a strong effect is when Peptides 7 and 8 are combined.

Peptides	1	2	3	4	5	6	7	8	9	10	11	12
1		++++	++++	−	−	−	−	−	−	+	−	++++
2	++++		++++	++++	+++	++++	++++	++++	+++	++++	++++	++++
3	++++	++++		++++	++++	+++	++++	++++	++++	+++	++++	++++
4	−	++++	++++		−	−	−	−	−	+	−	++++
5	−	+++	++++	−		+	−	−	−	−	+	++++
6	−	+++	++	−	+		+	−	−	−	+	++++
7	−	++++	++++	+	−	+		+++	++	-	+	++++
8	+	++++	++++	−	−	−	+++		+	−	−	++++
9	−	+++	++++	−	−	−	++	+		−	−	++++
10	+	+++	++	+	−	−	−	−	−		−	++++
11	−	++++	++++	−	+	−	+	−	−	−		++++
12	++++	+++	++++	++++	++++	++++	++++	++++	++++	++++	++++	

## Data Availability

The original contributions presented in this study are included in the article/Supplementary Materials. Further inquiries can be directed to the corresponding author.

## Supplementary Materials

The following supporting information can be downloaded at https://www.mdpi.com/article/10.3390/molecules30183790/s1, Figure S1: Synergistic effects of pairwise combinations of twelve CED4-derived peptides on *Caenorhabditis elegans* mortality and apoptosis at a total concentration of 0.4 mg/mL; Figure S2: Synergistic effects of pairwise combinations of twelve CED4-derived peptides on *Caenorhabditis elegans* mortality and apoptosis at a total concentration of 0.8 mg/mL; Figure S3. Three-dimensional structure predictions of the peptides used. These predictions were generated using PEP-FOLD3 [32].
