# Supplementary material for: CED4 and CED4-like Peptides as Effective Plant Parasitic Nematicides
Source: Molecules. 2025 Sep 18;30(18):3790. doi: 10.3390/molecules30183790 (PMC12472500; doi:10.3390/molecules30183790)

Figure S3. 3D structure predictions of the peptides used. These predictions were generated using PEP-FOLD3 [32].

Peptide Name and AA sequence	*3D structure prediction*
**Peptide 1** (aa residues 80-97 from CED4):<br>Q S H L A D F L E D Y I D F A I N E* (18)	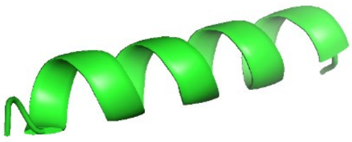
**Peptide 2** (aa residues 99-113 from CED4):<br>M D L L R P V V I A P Q F S R Q * (15)	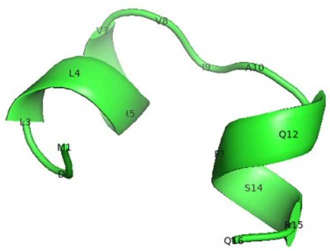
**Peptide 2a** (aa residues 99-113 from CED4 with two changes in red):<br>M E L L R P V V I A P Q F S R E * (15)	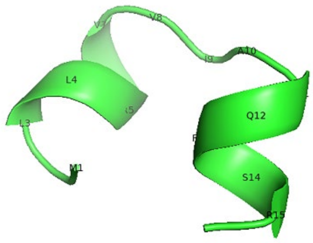
**Peptide 3** (aa residues 112-130 from CED4):<br>M R Q M L D R K L L L G N V P K Q M T C *<br>(19)	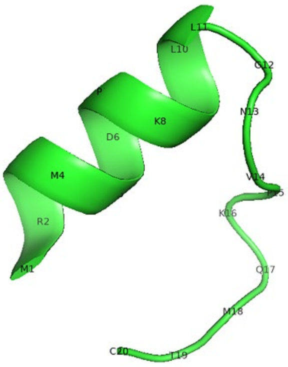
**Peptide 3a** (aa residues 112-130 from CED4 with two changes in red):<br>M K E M L D R K L L L G N V P K Q M T C *<br>(19)	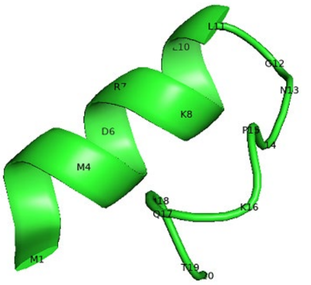

Peptide 4 (aa residues 129-141 from CED4):
T C Y I R E Y H V D R V I * (13)

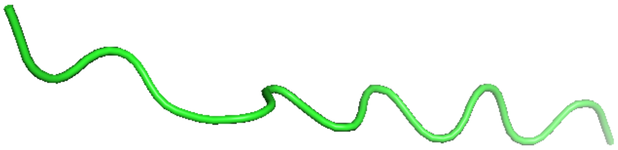

Peptide 5 (aa residues 207-220 from CED4):
I L L M L K S E D D L L N F* (14)

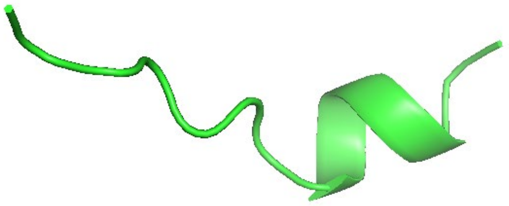

Peptide 6 (aa residues 227-240 from CED4):
M T S V V L K R M I **C** N A L I * (14)

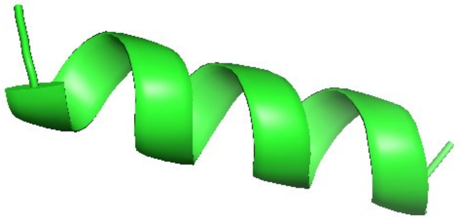

Peptide 7 (aa residues 274-294 from CED4):
D V E I S N A A S Q T C E F I E V T S L E* (21)

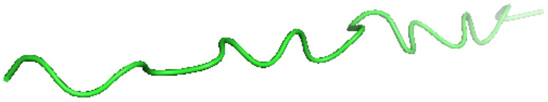

Peptide 8 (aa residues 334-358 from CED4):
M M F F K S C E P K T F E K M A Q L N N K L
E S R* (25)

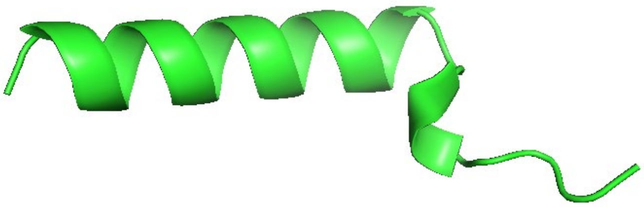

Peptide 9 (aa residues 360-380 from CED4):
L V G V E C I T P Y S Y K S L A M A L Q R*
(21)

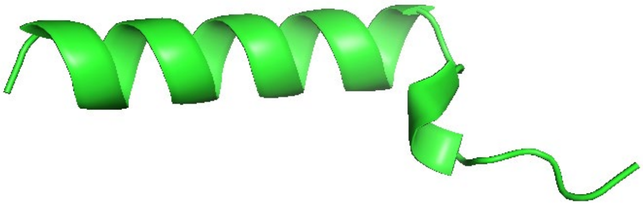

Peptide 10 (aa residues 442-456 from CED4):
A L L S G K R M P V L T F K I* (15)

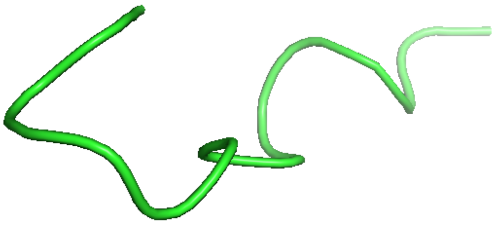

Peptide 11 (aa residues 468-484 from CED4):
V D A Q T I A N G I S I L E Q R L* (17)

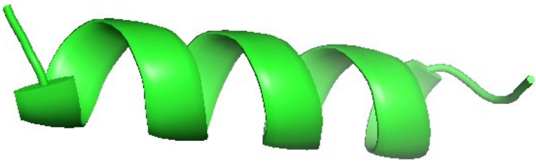

Peptide 12 (aa residues 529-540 from CED4):
M F P K F M Q L H Q K F Y * (12)

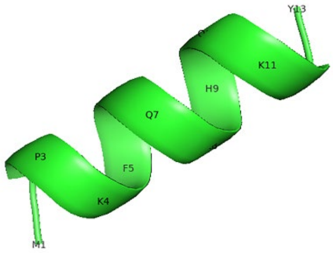

Peptide 12a (aa residues 529-540 from CED4
with two changes in red):
M Y P K F M Q L H Q K F F * (12)

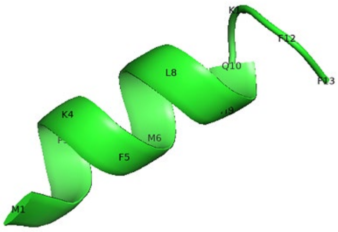

Supplement: Supplementary file 1 [file molecules-30-03790-s001.zip › Figure S3 Ribbon Struc.pdf]
