# Supplementary material for: CED4 and CED4-like Peptides as Effective Plant Parasitic Nematicides
Source: Molecules. 2025 Sep 18;30(18):3790. doi: 10.3390/molecules30183790 (PMC12472500; doi:10.3390/molecules30183790)
Supplement: Supplementary file 1 [file molecules-30-03790-s001.zip › Figures S1 and S2.pdf]

Figure S1. Synergistic effects of pairwise combinations of twelve CED4-derived peptides on *Caenorhabditis elegans* mortality and apoptosis at a total concentration of **0.4 mg/mL**.

This figure presents the results of 66 pairwise combinations of twelve synthetic peptides derived from the *C. elegans* CED-4 protein, each tested at a total concentration of **0.4 mg/mL** to evaluate synergistic nematocidal activity. Mortality and apoptosis were assessed over a 96-hour period. Panel (**a**) shows that Peptide 1, when combined with Peptides 2, 3, or 12, induced significant mortality, with the Peptide 1 + 2 combination achieving nearly 60% lethality. Peptides 4 and 9, despite being categorized as negative peptides, contributed to a minor mortality rate when combined with Peptide 1.

In panel (**b**), Peptide 2 demonstrated enhanced efficacy in combination with several other peptides, inducing up to 20% apoptosis within 96 hours. The Peptide 2 + 3 combination exhibited the highest activity. Panel (**c**) shows that other combinations also induced >30% apoptosis by 96 hours, although none were effective within the first 24 hours. Panel (**d**) indicates that Peptide 4 paired with Peptides 2, 3, or 12 yielded >30% mortality, while combinations with Peptides 5, 6, 8, and 9 were ineffective. Peptide 5 (panel **e**) showed generally low efficacy, with minor apoptosis when combined with Peptides 7, 9, 10, and 11.

Panel (**f**) demonstrates the utility of Peptide 6 as a negative control, showing negligible mortality or apoptosis even in combination with other peptides. In panel (*g*), Peptide 7 displayed weak activity, with slight effects noted in combinations with positive peptides. Peptide 8 (panel **h**) showed ≥30% apoptosis by 96 hours in combination with Peptides 2, 3, and 12. Panels (**i**) and (**j**) reveal that Peptides 9 and 10, respectively, displayed limited activity, with Peptide 9 more effective when combined with Peptide 12. Panel (**k**) shows Peptide 11's synergistic potential in all pairings except with Peptide 1, while Peptide 12 (panel **l**) exhibited strong synergistic efficacy in all tested combinations, inducing >40% mortality by Day 4.

These results collectively suggest that specific combinations of CED4-derived peptides, particularly those involving Peptides 2, 3, and 12, can enhance nematocidal effects via activation of programmed cell death pathways in *C. elegans*.

a

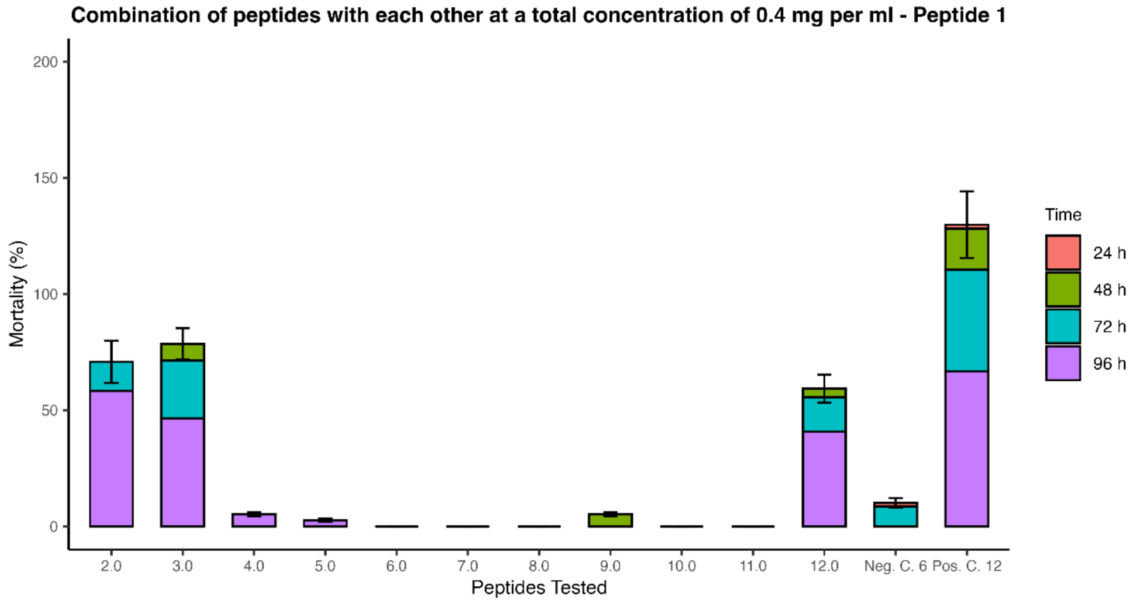

Combination of peptides with each other at a total concentration of 0.4 mg per ml - Peptide 1
Mortality (%)
200
150
100
50
0
2.0
3.0
4.0
5.0
6.0
7.0
8.0
9.0
10.0
11.0
12.0
Neg. C. 6
Pos. C. 12
Peptides Tested
Time
24 h
48 h
72 h
96 h

b

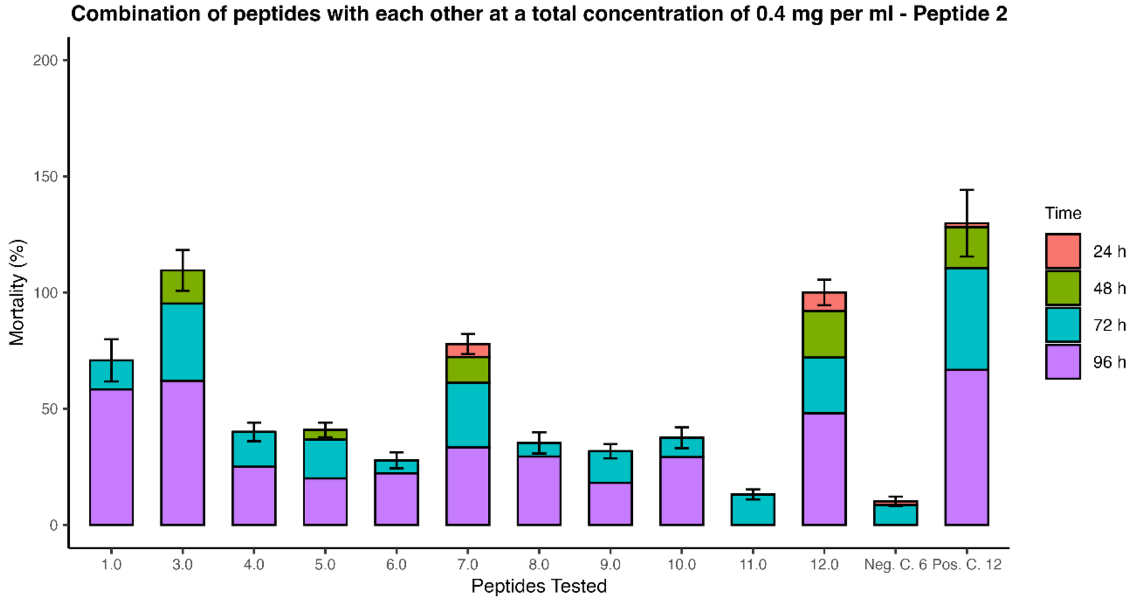

Combination of peptides with each other at a total concentration of 0.4 mg per ml - Peptide 2
Mortality (%)
200
150
100
50
0
1.0
3.0
4.0
5.0
6.0
7.0
8.0
9.0
10.0
11.0
12.0
Neg. C. 6
Pos. C. 12
Peptides Tested
Time
24 h
48 h
72 h
96 h

c

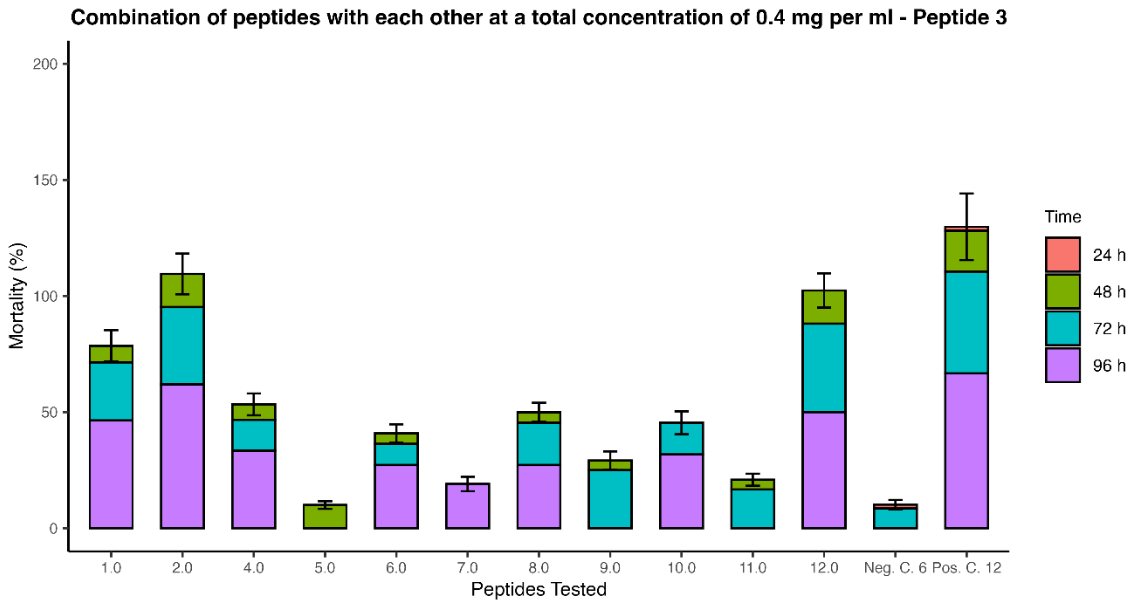
Combination of peptides with each other at a total concentration of 0.4 mg per ml - Peptide 3
Mortality (%)
200
150
100
50
0
1.0
2.0
4.0
5.0
6.0
7.0
8.0
9.0
10.0
11.0
12.0
Neg. C. 6
Pos. C. 12
Peptides Tested
Time
24 h
48 h
72 h
96 h

d

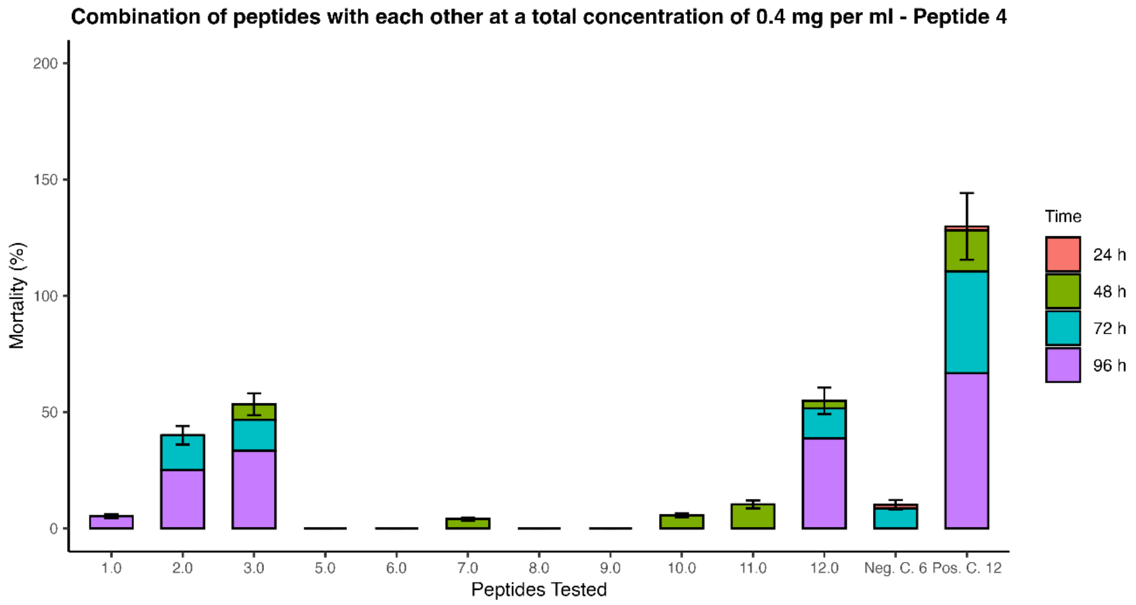
Combination of peptides with each other at a total concentration of 0.4 mg per ml - Peptide 4
Mortality (%)
200
150
100
50
0
1.0
2.0
3.0
5.0
6.0
7.0
8.0
9.0
10.0
11.0
12.0
Neg. C. 6
Pos. C. 12
Peptides Tested
Time
24 h
48 h
72 h
96 h

e

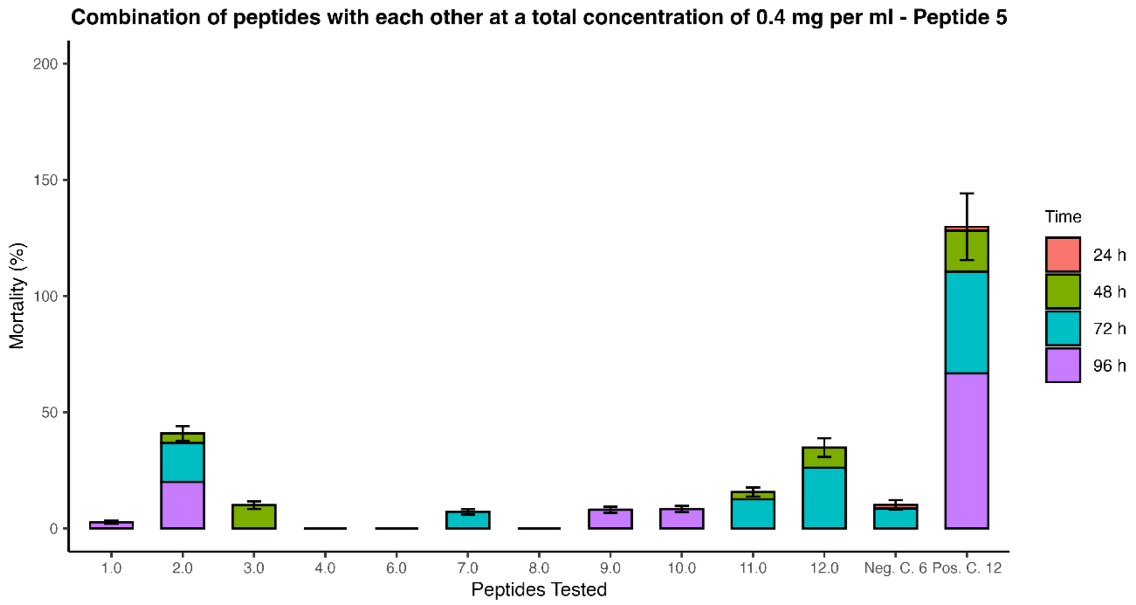
Combination of peptides with each other at a total concentration of 0.4 mg per ml - Peptide 5
Mortality (%)
200
150
100
50
0
1.0
2.0
3.0
4.0
6.0
7.0
8.0
9.0
10.0
11.0
12.0
Neg. C. 6
Pos. C. 12
Peptides Tested
Time
24 h
48 h
72 h
96 h

f

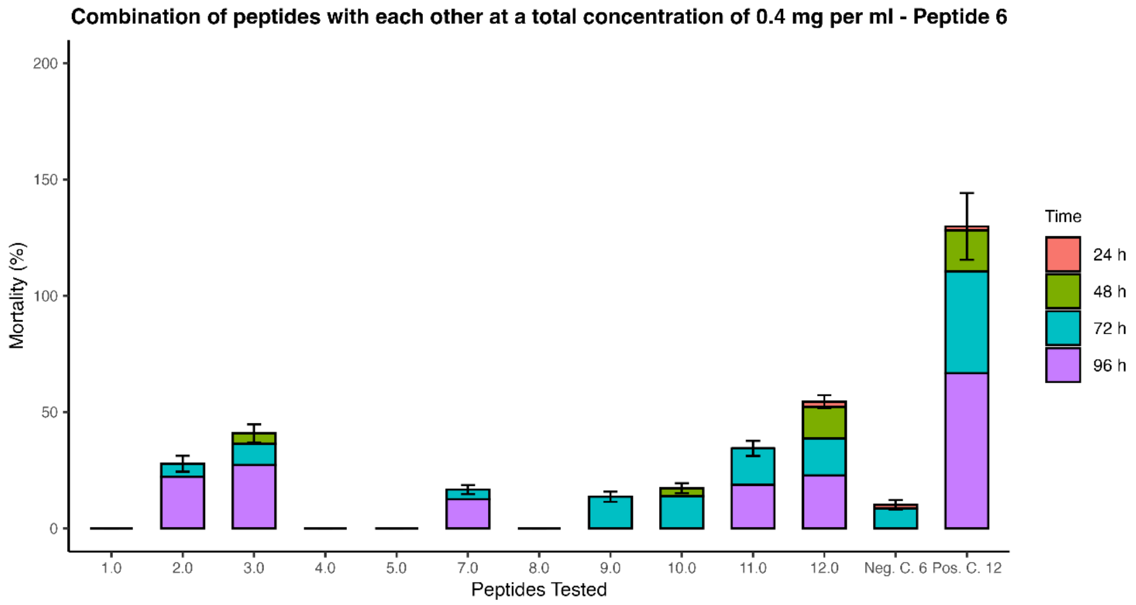
Combination of peptides with each other at a total concentration of 0.4 mg per ml - Peptide 6
Mortality (%)
200
150
100
50
0
1.0
2.0
3.0
4.0
5.0
7.0
8.0
9.0
10.0
11.0
12.0
Neg. C. 6
Pos. C. 12
Peptides Tested
Time
24 h
48 h
72 h
96 h

g

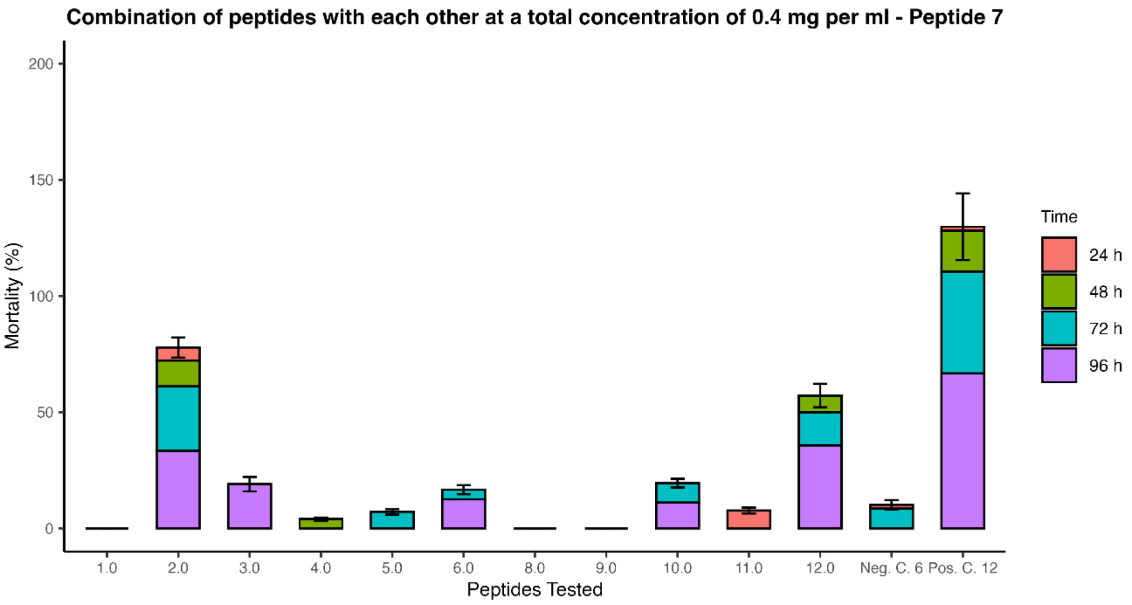
Combination of peptides with each other at a total concentration of 0.4 mg per ml - Peptide 7
Mortality (%)
200
150
100
50
0
1.0
2.0
3.0
4.0
5.0
6.0
8.0
9.0
10.0
11.0
12.0
Neg. C. 6
Pos. C. 12
Peptides Tested
Time
24 h
48 h
72 h
96 h

h

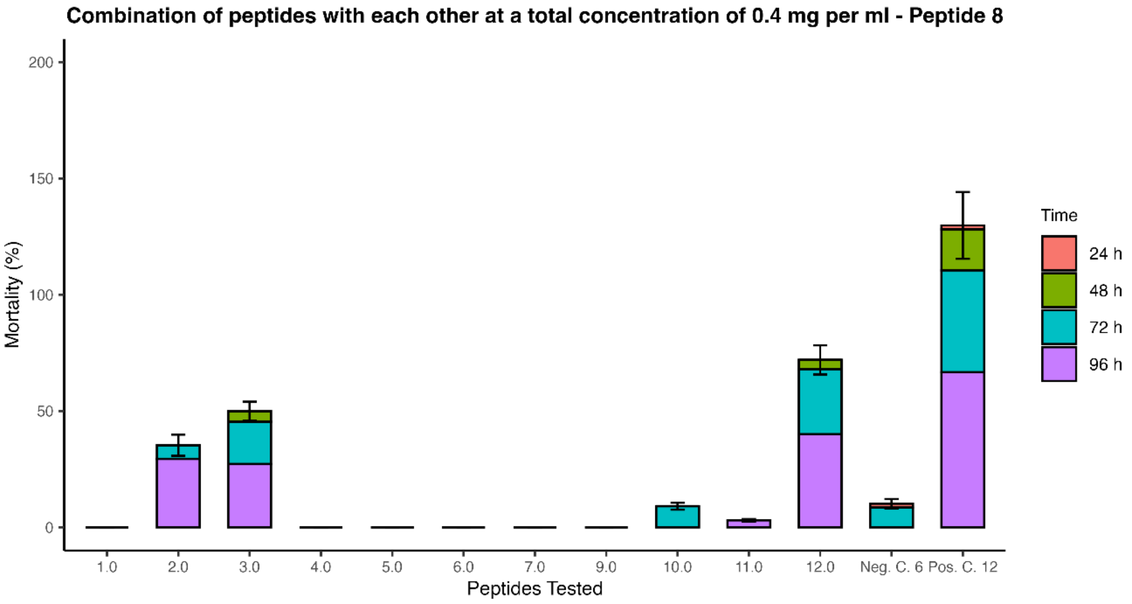
Combination of peptides with each other at a total concentration of 0.4 mg per ml - Peptide 8
Mortality (%)
200
150
100
50
0
1.0
2.0
3.0
4.0
5.0
6.0
7.0
9.0
10.0
11.0
12.0
Neg. C. 6
Pos. C. 12
Peptides Tested
Time
24 h
48 h
72 h
96 h

i

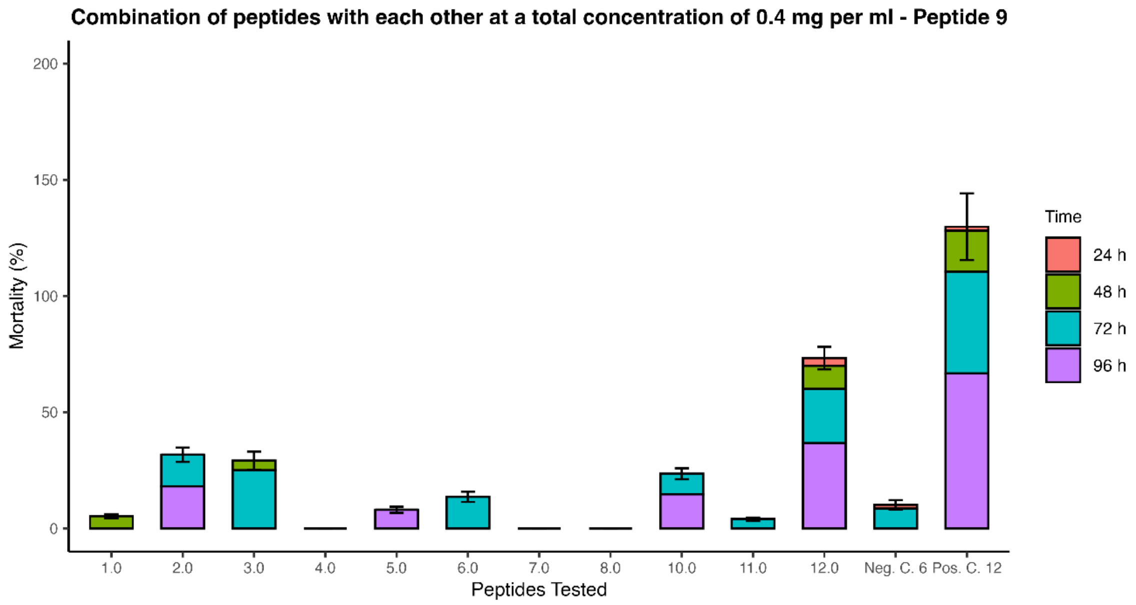

Combination of peptides with each other at a total concentration of 0.4 mg per ml - Peptide 9
Mortality (%)
200
150
100
50
0
1.0
2.0
3.0
4.0
5.0
6.0
7.0
8.0
10.0
11.0
12.0
Neg. C. 6
Pos. C. 12
Peptides Tested
Time
24 h
48 h
72 h
96 h

j

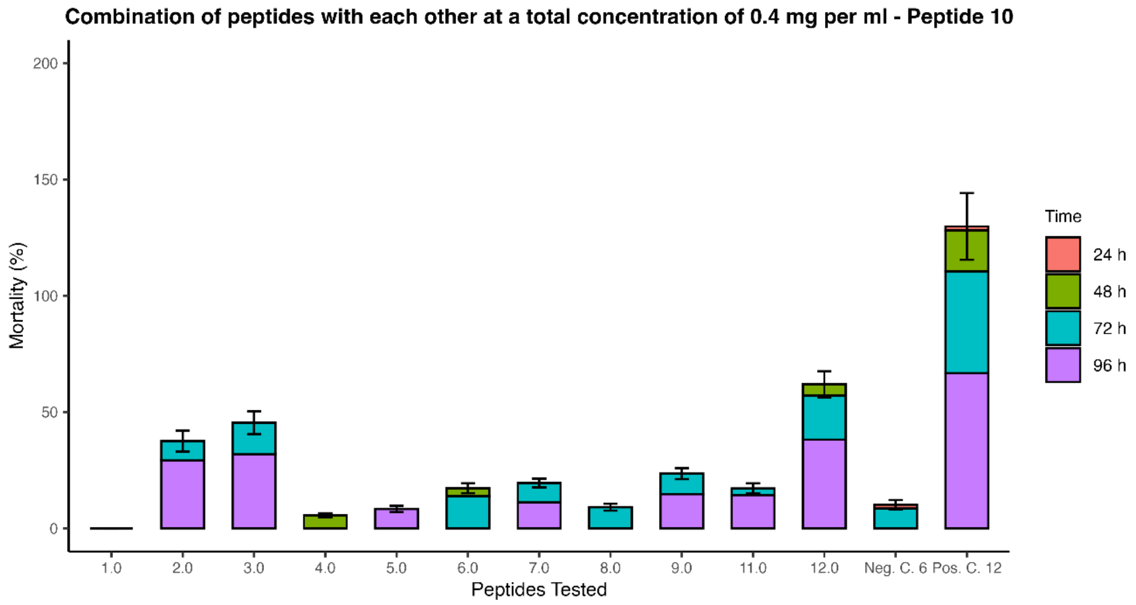

Combination of peptides with each other at a total concentration of 0.4 mg per ml - Peptide 10
Mortality (%)
200
150
100
50
0
1.0
2.0
3.0
4.0
5.0
6.0
7.0
8.0
9.0
11.0
12.0
Neg. C. 6
Pos. C. 12
Peptides Tested
Time
24 h
48 h
72 h
96 h

k

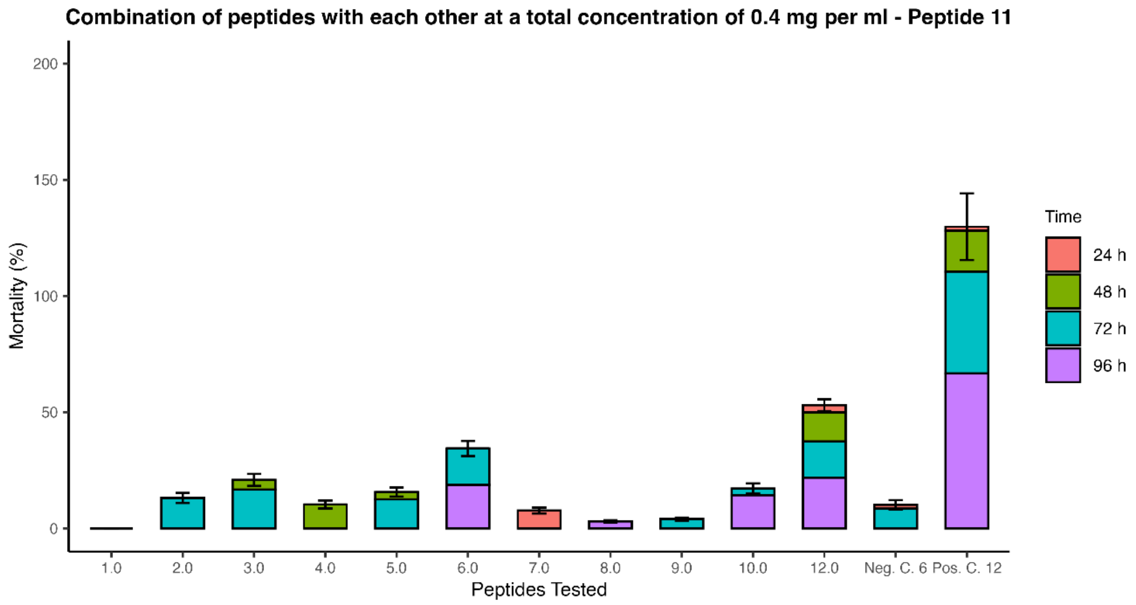

Combination of peptides with each other at a total concentration of 0.4 mg per ml - Peptide 11
Mortality (%)
200
150
100
50
0
1.0
2.0
3.0
4.0
5.0
6.0
7.0
8.0
9.0
10.0
12.0
Neg. C. 6
Pos. C. 12
Peptides Tested
Time
24 h
48 h
72 h
96 h

l

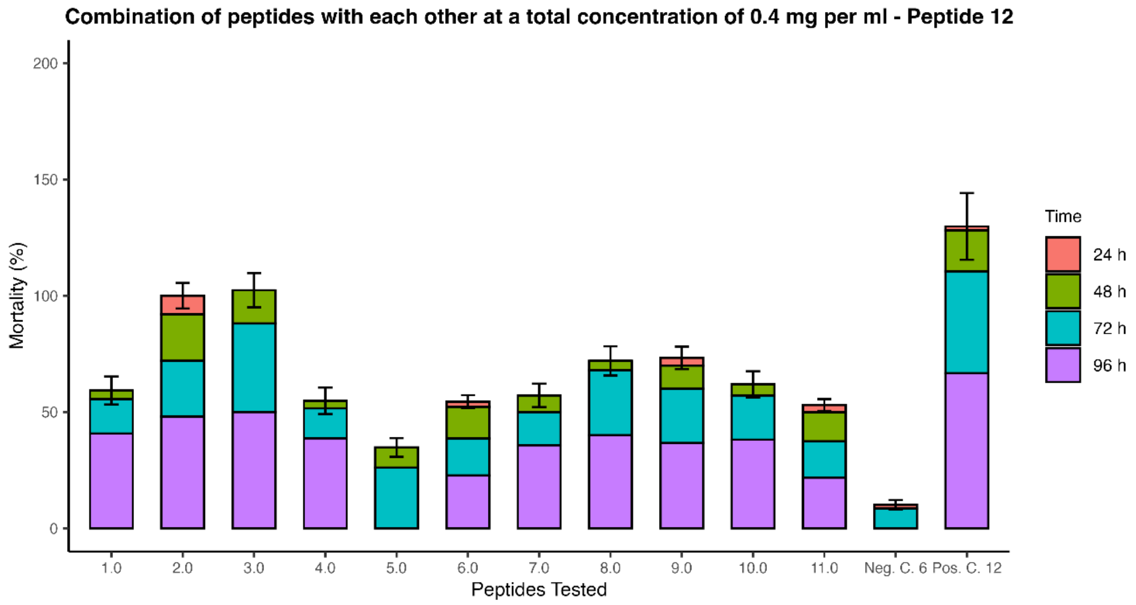

Combination of peptides with each other at a total concentration of 0.4 mg per ml - Peptide 12
Mortality (%)
200
150
100
50
0
1.0
2.0
3.0
4.0
5.0
6.0
7.0
8.0
9.0
10.0
11.0
Neg. C. 6
Pos. C. 12
Peptides Tested
Time
24 h
48 h
72 h
96 h

Figure S2. Synergistic effects of pairwise combinations of twelve CED4-derived peptides on *Caenorhabditis elegans* mortality and apoptosis at a total concentration of **0.8 mg/mL**.

This figure illustrates the nematocidal activity of 66 unique pairwise combinations of twelve synthetic peptides derived from the *C. elegans* CED-4 protein, tested at an elevated total concentration of **0.8 mg/mL**. Each peptide was evaluated in binary combinations to assess synergistic enhancement in inducing programmed cell death. Mortality and apoptosis were measured over a 96-hour period.

Panel (**a**) demonstrates that Peptide 1 exhibited strong efficacy when combined with Peptides 2, 3, and 12, achieving complete (100%) mortality by Day 4, particularly with Peptide 2. Additional minor mortality effects were observed with combinations involving Peptides 8 and 10. Peptides 4 and 9, typically classified as negative controls, also showed >5% lethality when combined with Peptide 1. In panel (**b**), Peptide 2 demonstrated consistent synergistic effects across all combinations, with average mortality rates exceeding 70%. Peptide 3 (panel **c**) also achieved 100% mortality when combined with Peptides 1, 2, 4, 5, 7, 9, and 12, and induced >60% mortality even when paired with negative peptides such as Peptides 6 and 10.

Panel (**d**) indicates that Peptide 4 combined with Peptides 2, 3, and 12 led to complete mortality by Day 4, with the Peptide 4 + 12 combination inducing death within 72 hours. Panel (**e**) reveals that Peptide 5 demonstrated maximal lethality only in combination with Peptides 3 and 12, while minimal apoptosis was observed with combinations involving Peptides 6 and 11.

Panels (**f**) through (**h**) highlight moderate efficacy of Peptides 6, 7, and 8 in specific combinations. Peptide 6 induced apoptosis when paired with Peptides 2 and 3 but otherwise showed limited activity. Peptide 7, when combined with Peptides 2 and 3, resulted in greater efficacy than with other combinations, and induced >25% apoptosis with Peptides 4, 6, 8, 9, and 11. Peptide 8 demonstrated enhanced lethality when paired with Peptides 2, 3, and 12, and modest mortality (~20%) when combined with negative peptides such as Peptides 7 and 9 (panels **h**–**i**).

Panel (**j**) shows that Peptide 10 combinations generally exhibited weak activity, with <10% mortality observed in most pairings. Peptide 11 (panel **k**) demonstrated higher efficacy when combined with Peptides 2, 3, and 12 (up to 70% mortality), but lower mortality (~10%) with Peptides 5, 6, and 7. Panel (**l**) illustrates that Peptide 12 exerted potent synergistic effects when paired with Peptide 3, achieving nearly 100% lethality in 72 hours. Peptide 12 combinations with other peptides—including Peptides 1, 4, 5, 6, 7, 8, and 11—also exhibited >80% mortality.

Overall, these results confirm that specific combinations, particularly those involving Peptides 2, 3, and 12, significantly enhance programmed cell death in *C. elegans* at elevated concentrations, supporting their potential use as synergistic nematocidal agents.

a

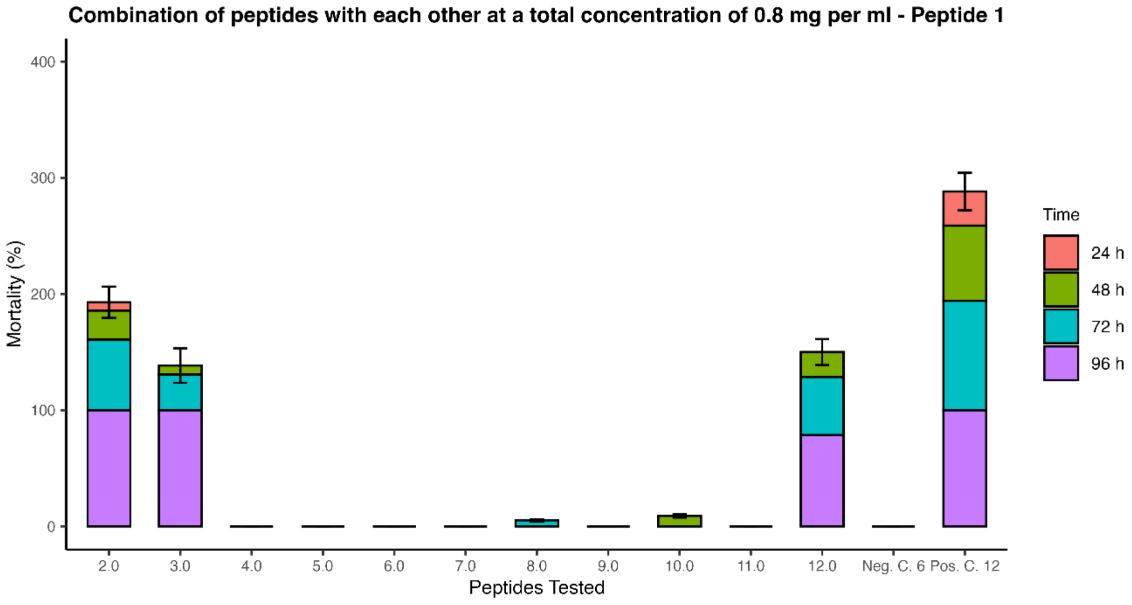
Combination of peptides with each other at a total concentration of 0.8 mg per ml - Peptide 1
Mortality (%)
400
300
200
100
0
2.0
3.0
4.0
5.0
6.0
7.0
8.0
9.0
10.0
11.0
12.0
Neg. C. 6
Pos. C. 12
Peptides Tested
Time
24 h
48 h
72 h
96 h

b

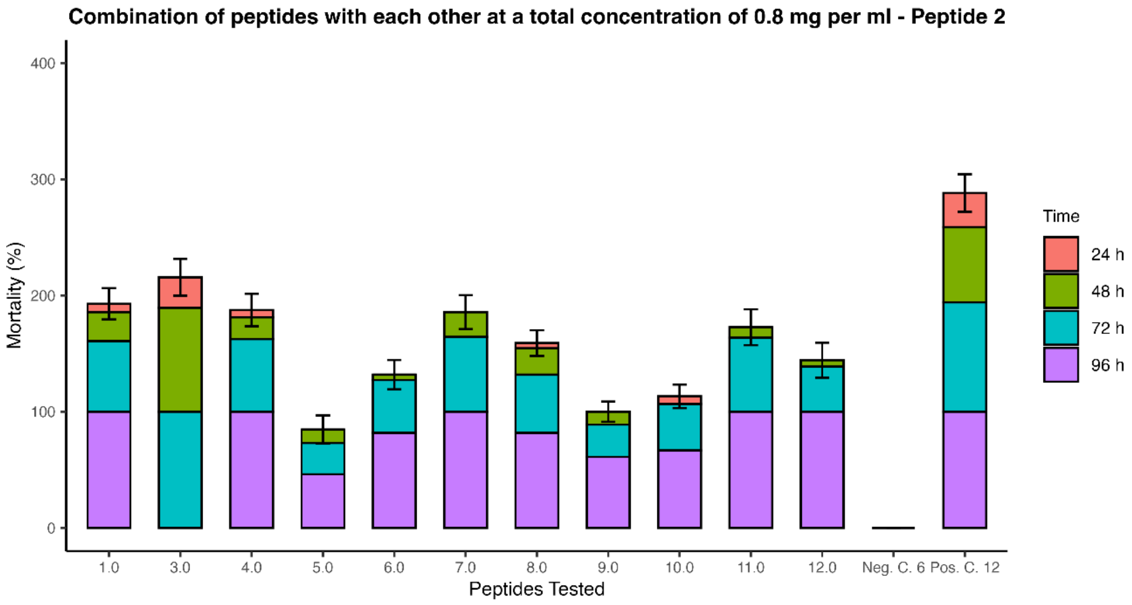
Combination of peptides with each other at a total concentration of 0.8 mg per ml - Peptide 2
Mortality (%)
400
300
200
100
0
1.0
3.0
4.0
5.0
6.0
7.0
8.0
9.0
10.0
11.0
12.0
Neg. C. 6
Pos. C. 12
Peptides Tested
Time
24 h
48 h
72 h
96 h

c

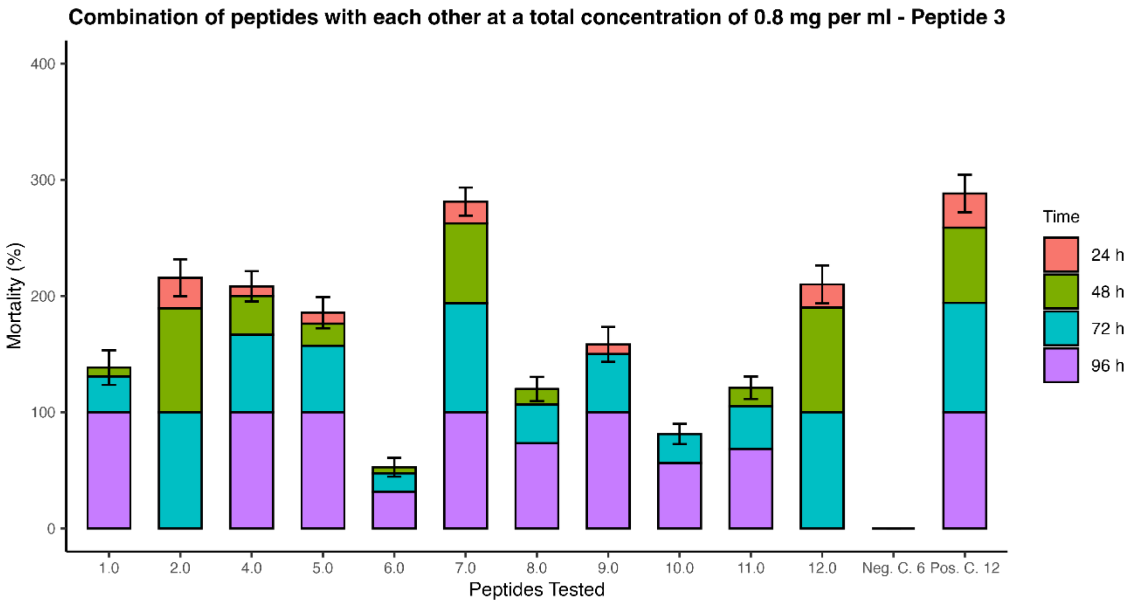

Combination of peptides with each other at a total concentration of 0.8 mg per ml - Peptide 3
Mortality (%)
0
100
200
300
400
1.0
2.0
4.0
5.0
6.0
7.0
8.0
9.0
10.0
11.0
12.0
Neg. C. 6
Pos. C. 12
Peptides Tested
Time
24 h
48 h
72 h
96 h

d

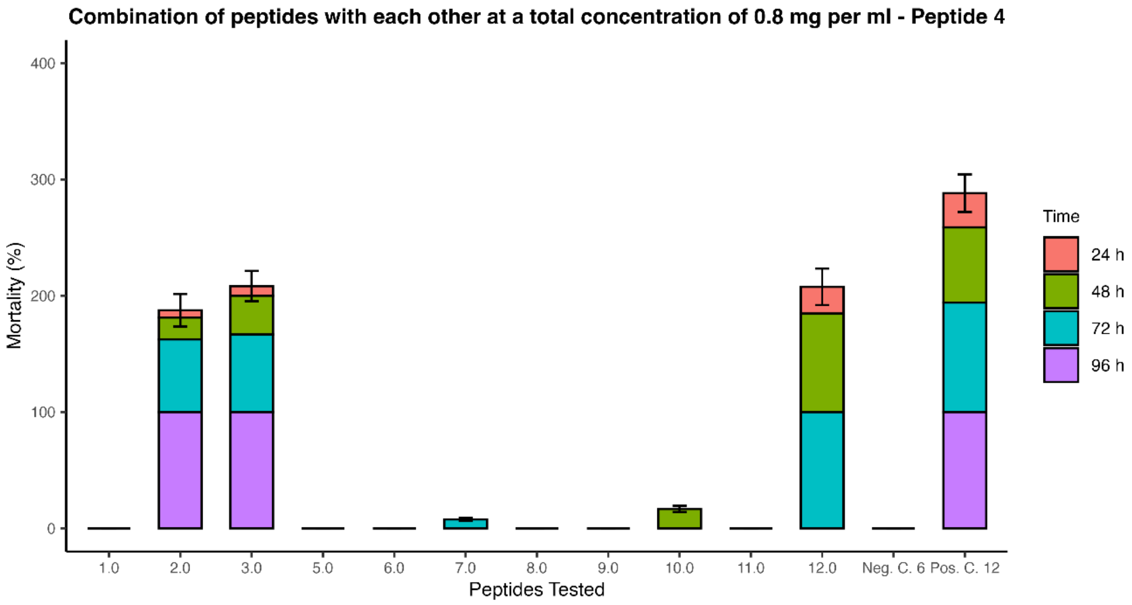

Combination of peptides with each other at a total concentration of 0.8 mg per ml - Peptide 4
Mortality (%)
0
100
200
300
400
1.0
2.0
3.0
5.0
6.0
7.0
8.0
9.0
10.0
11.0
12.0
Neg. C. 6
Pos. C. 12
Peptides Tested
Time
24 h
48 h
72 h
96 h

e

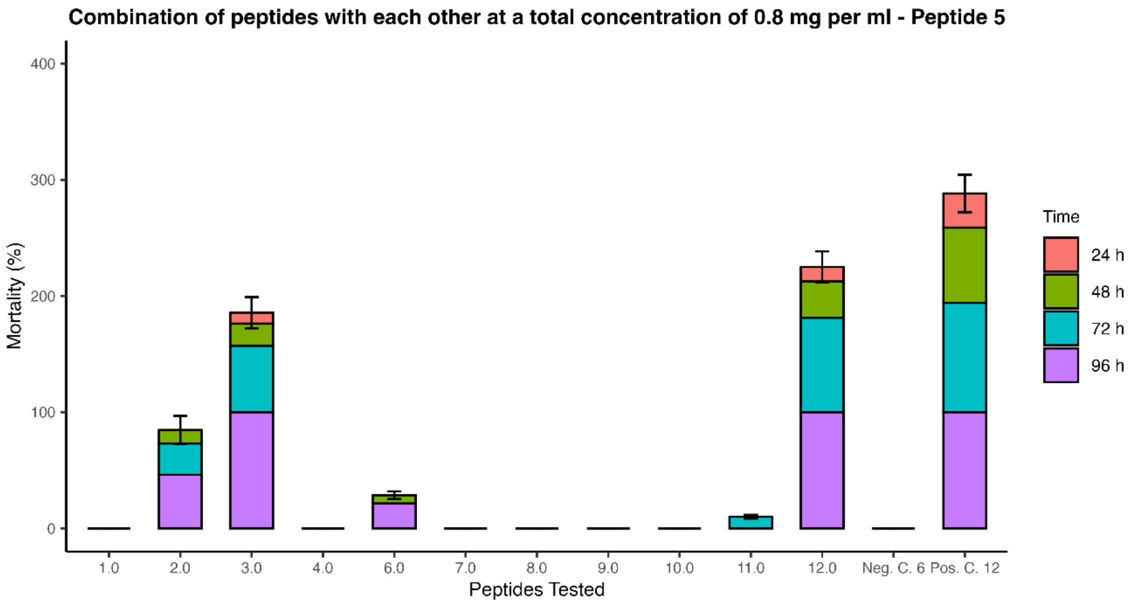

Combination of peptides with each other at a total concentration of 0.8 mg per ml - Peptide 5
Mortality (%)
400
300
200
100
0
1.0
2.0
3.0
4.0
6.0
7.0
8.0
9.0
10.0
11.0
12.0
Neg. C. 6
Pos. C. 12
Peptides Tested
Time
24 h
48 h
72 h
96 h

f

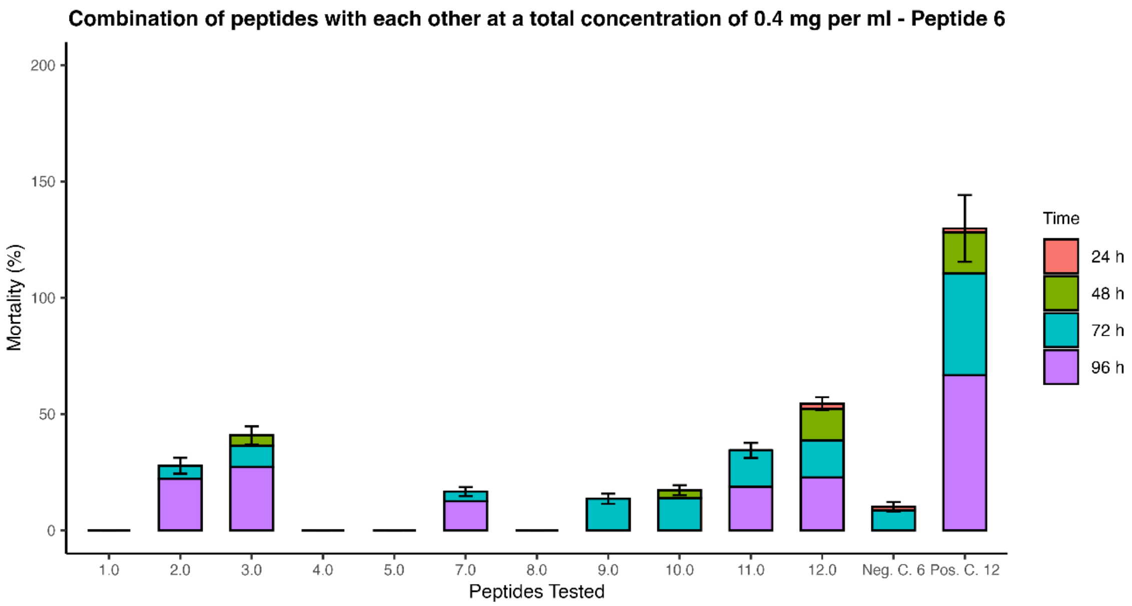

Combination of peptides with each other at a total concentration of 0.4 mg per ml - Peptide 6
Mortality (%)
200
150
100
50
0
1.0
2.0
3.0
4.0
5.0
7.0
8.0
9.0
10.0
11.0
12.0
Neg. C. 6
Pos. C. 12
Peptides Tested
Time
24 h
48 h
72 h
96 h

g

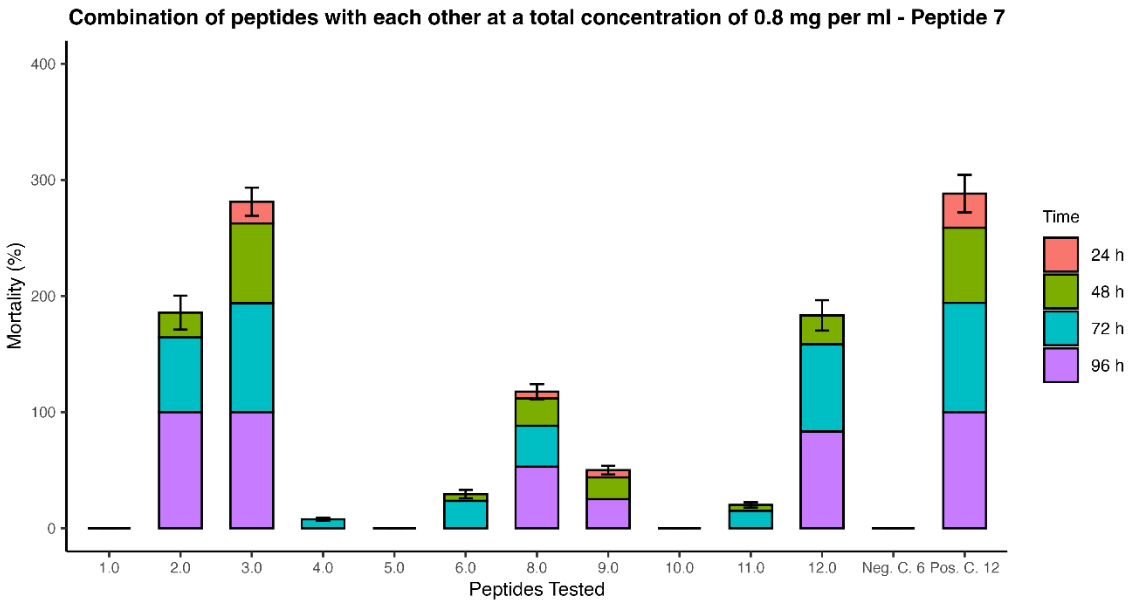
Combination of peptides with each other at a total concentration of 0.8 mg per ml - Peptide 7
Mortality (%)
400
300
200
100
0
1.0
2.0
3.0
4.0
5.0
6.0
8.0
9.0
10.0
11.0
12.0
Neg. C. 6
Pos. C. 12
Peptides Tested
Time
24 h
48 h
72 h
96 h

h

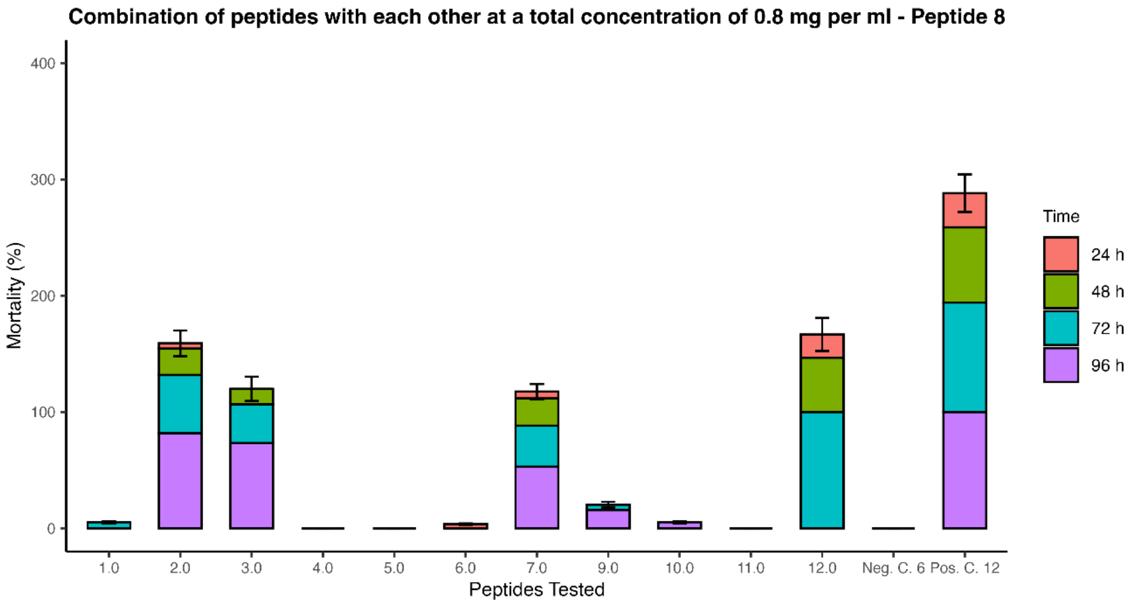
Combination of peptides with each other at a total concentration of 0.8 mg per ml - Peptide 8
Mortality (%)
400
300
200
100
0
1.0
2.0
3.0
4.0
5.0
6.0
7.0
9.0
10.0
11.0
12.0
Neg. C. 6
Pos. C. 12
Peptides Tested
Time
24 h
48 h
72 h
96 h

i

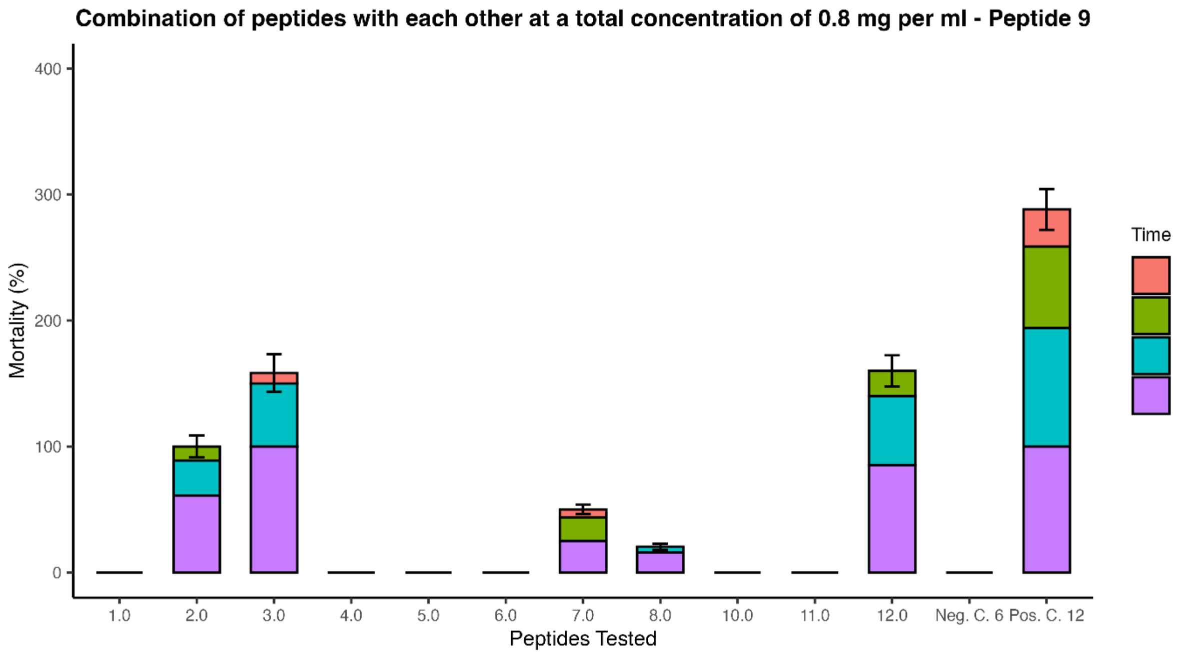
Combination of peptides with each other at a total concentration of 0.8 mg per ml - Peptide 9
Mortality (%)
0
100
200
300
400
1.0
2.0
3.0
4.0
5.0
6.0
7.0
8.0
10.0
11.0
12.0
Neg. C. 6
Pos. C. 12
Peptides Tested
Time

j

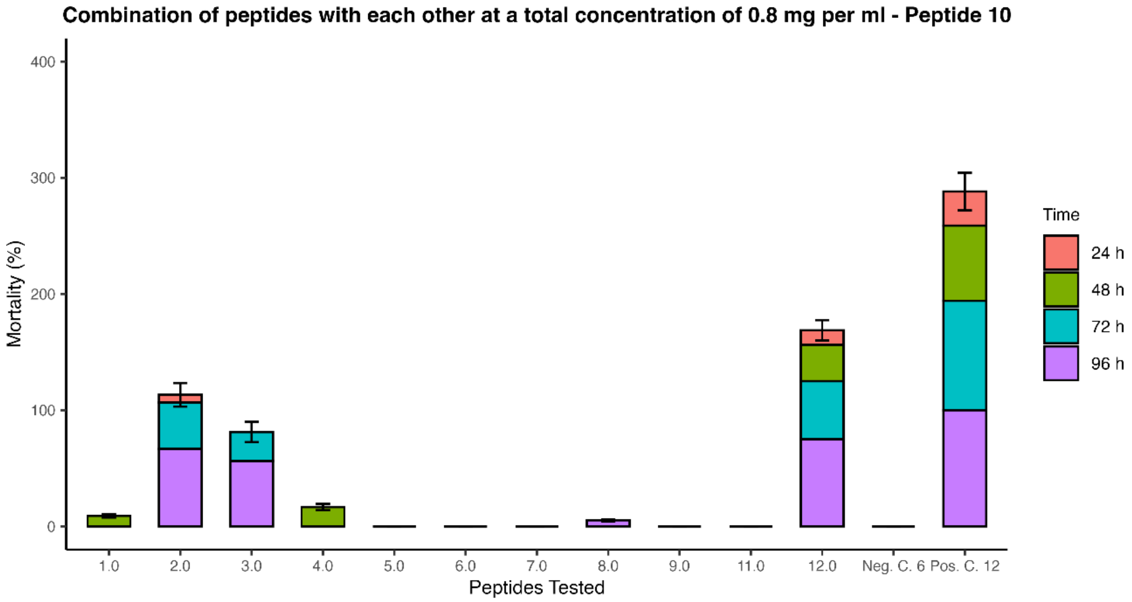
Combination of peptides with each other at a total concentration of 0.8 mg per ml - Peptide 10
Mortality (%)
0
100
200
300
400
1.0
2.0
3.0
4.0
5.0
6.0
7.0
8.0
9.0
11.0
12.0
Neg. C. 6
Pos. C. 12
Peptides Tested
Time
24 h
48 h
72 h
96 h

k

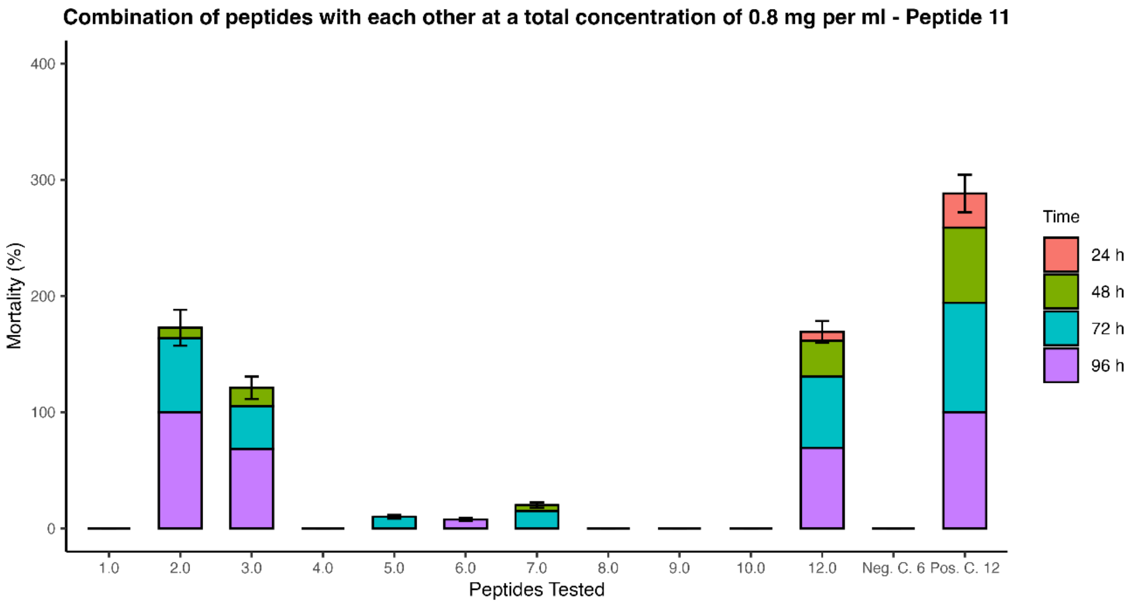

Combination of peptides with each other at a total concentration of 0.8 mg per ml - Peptide 11
Mortality (%)
0
100
200
300
400
1.0
2.0
3.0
4.0
5.0
6.0
7.0
8.0
9.0
10.0
12.0
Neg. C. 6
Pos. C. 12
Peptides Tested
Time
24 h
48 h
72 h
96 h

l

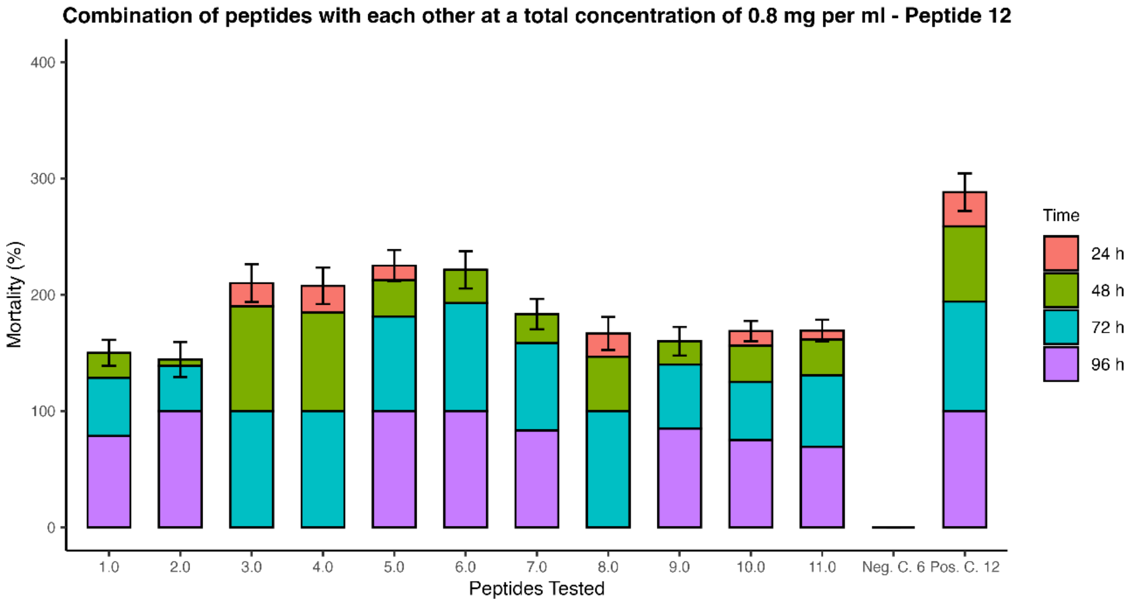

Combination of peptides with each other at a total concentration of 0.8 mg per ml - Peptide 12
Mortality (%)
0
100
200
300
400
1.0
2.0
3.0
4.0
5.0
6.0
7.0
8.0
9.0
10.0
11.0
Neg. C. 6
Pos. C. 12
Peptides Tested
Time
24 h
48 h
72 h
96 h
